# Evolution of Hybrid Inviability Associated With Chromosome Fusions

**DOI:** 10.1111/mec.17672

**Published:** 2025-02-03

**Authors:** Jesper Boman, Karin Näsvall, Roger Vila, Christer Wiklund, Niclas Backström

**Affiliations:** ^1^ Evolutionary Biology Program, Department of Ecology and Genetics (IEG) Uppsala University Uppsala Sweden; ^2^ Institut de Biologia Evolutiva (CSIC‐Universitat Pompeu Fabra) Barcelona Spain; ^3^ Department of Zoology: Division of Ecology Stockholm University Stockholm Sweden

**Keywords:** chromosomal rearrangements, hybrid incompatibilities, hybrid inviability, population genomics, speciation

## Abstract

Chromosomal rearrangements, such as inversions, have received considerable attention in the speciation literature due to their hampering effects on recombination. Less is known about how other rearrangements, such as chromosome fissions and fusions, can affect the evolution of reproductive isolation. Here, we use crosses between populations of the wood white butterfly (*Leptidea sinapis*) with different karyotypes to identify genomic regions associated with hybrid inviability. We map hybrid inviability candidate loci by contrasting allele frequencies between F_2_ hybrids that survived until the adult stage with individuals of the same cohort that succumbed to hybrid incompatibilities. Hybrid inviability candidate regions have high genetic differentiation between parental populations, reduced recombination rates, and are enriched near chromosome fusions. By analysing sequencing coverage, we exclude aneuploidies as a direct link between hybrid inviability and chromosome fusions. Instead, our results point to an indirect relationship between hybrid inviability and chromosome fusions, possibly related to reduced recombination in fused chromosomes. Thus, we map postzygotic isolation to chromosomal rearrangements, providing crucial empirical evidence for the idea that chromosome number differences between taxa can contribute to speciation.

## 1 Introduction

Understanding the genetic underpinnings of speciation lies at the heart of evolutionary biology (Coyne and Orr 2004). A crucial aspect of the speciation process is how barriers to gene flow are established, since most novel species form as a consequence of reduced gene flow between populations within species (Coyne and Orr 2004; Wu 2001). One such barrier is hybrid inviability, the reduced survival of hybrid offspring. Crossing efforts in *Drosophila* and other organisms have shown that hybrid inviability generally conforms to the Bateson‐Dobzhansky‐Muller (BDMI) model, i.e., that alleles at two or more interacting genes are required for incompatibilities to manifest in hybrids (Barbash et al. 2003; Gadau, Page, and Werren 1999; Orr 2005). Nevertheless, genic interaction is not the only type of hybrid incompatibility. Also, chromosomal rearrangements such as polyploidizations, gene duplications, and inversions may form the genetic basis of hybrid incompatibilities. In addition, chromosomal rearrangements resulting in underdominant karyotypes may underlie hybrid sterility (e.g., King 1993; White 1968; Yoshida et al. 2023). However, the underdominance model has been criticised due to the limited parameter range (e.g., extremely small population size) under which it can evolve (Futuyma and Mayer 1980; Nei, Maruyama, and Wu 1983; Templeton 1981). While mainly associated with hybrid sterility (Berdan et al. 2023; Hauffe and Searle 1998; Pavlova and Searle 2018), chromosomal rearrangements can also cause hybrid inviability (Baker and Bickham 1986; King 1993). Non‐disjunction in either mitosis or F_1_ hybrid meiosis may cause aneuploidies that lead to embryonic inviability (Gibeaux et al. 2018). This would constitute a direct effect of chromosomal rearrangements on hybrid inviability. Chromosomal rearrangements may also contribute indirectly to speciation as a consequence of effects on the recombination rate, allowing for co‐inheritance of alleles at different sites in the face of gene flow (Faria and Navarro 2010; Noor et al. 2001; Rafajlović et al. 2021; Rieseberg 2001).

Based on whether chromosomal rearrangements reduce recombination in heterokaryotypes, homokaryotypes, or both, they can be divided into two different categories: (i) Rearrangements that reduce recombination only in heterokaryotypes may promote divergent evolution of genes located within the rearranged region, which can lead to the evolution of reproductive isolation (Butlin 2005; Faria and Navarro 2010; Kirkpatrick and Barton 2006; Navarro and Barton 2003); (ii) Rearrangements that reduce the recombination rate in both hetero‐ and homokaryotypes can also promote the evolution of reproductive isolation and will lead to increased selection on linked sites by reducing the effective population size (*N*_e_) in the rearranged region. This leads to faster lineage sorting (Pease and Hahn 2013) and, consequently, shorter expected fixation times of segregating alleles (Kimura 1980). Regions with reduced recombination are also expected to accumulate less introgressed DNA (Aeschbacher et al. 2017; Barton and Bengtsson 1986; Veller et al. 2023) and harbour incompatibilities during more generations in cases of secondary contact between diverged populations (Rafajlović et al. 2021). Thus, several models predict that regions with low recombination rates have a higher probability of including loci associated with reproductive isolation. Previous theoretical and empirical work has predominantly focused on rearrangements that cause recombination suppression only in heterokaryotypes, such as inversions (Coyne and Orr 2004; Gong, McKim, and Hawley 2005; Kirkpatrick and Barton 2006; Noor et al. 2001; Rieseberg 2001; Sturtevant and Beadle 1936). Comparatively little is known about the consequences of chromosomal rearrangements that also reduce homokaryotype recombination, for example, chromosome fusions, which can cause cessation of recombination between loci near the fusion point that were previously segregating independently (Dumas and Britton‐Davidian 2002; Guerrero and Kirkpatrick 2014; Liu et al. 2022; Mackintosh, Vila, Laetsch et al. 2023; Näsvall et al. 2023; Ortiz‐Barrientos, Engelstädter, and Rieseberg 2016; Yoshida et al. 2023).

Here, we investigate the genomic basis of hybrid inviability among populations of the wood white butterfly (*Leptidea sinapis*) with distinct karyotypes, using sequencing of large sets of pooled individuals (PoolSeq). *Leptidea sinapis* is an excellent model system for studying the effects of chromosomal rearrangements on the evolution of hybrid inviability since it has the most extreme intraspecific chromosome number variation among all diploid eukaryotes (Lukhtanov et al. 2011). *Leptidea*, like all butterflies, have female heterogamety (ZW sex chromosomes), female achiasmy (no recombination in females), and holocentric chromosomes, meaning that centromere activity is dispersed along the chromosomes (Rosin et al. 2021; Šíchová et al. 2015). Cytogenetically confirmed chromosome numbers range from 2*n* = 57, 58 in Sweden (SWE) and 2*n* = 56–64 in Kazakhstan to 2*n* = 106, 108 in Catalonia (CAT; Lukhtanov et al. 2011, 2018). A cline in chromosome number stretches from Fennoscandia in the north and Kazakhstan to Iran in the east to the Iberian Peninsula in the southwest (Lukhtanov et al. 2011, Nazari et al. 2023). A recent comparative study revealed that the difference in karyotype structure between the SWE and CAT populations is a consequence of numerous fissions and fusions (Höök et al. 2023). Most of these fissions/fusions are simple rearrangements where SWE has one and CAT has two chromosomes, while others are more complex (sometimes called chain rearrangements) and involve several chromosomes (Höök et al. 2023). The wood white has previously been subject to studies on reproductive isolation since it is morphologically cryptic but differs in genital morphology and chromosome number from the congenerics *L. reali* and *L. juvernica* (Dincă et al. 2011, 2013). In addition, *L. sinapis* from SWE and CAT have been crossed to investigate reproductive isolation between these populations in general and the role of the fissions and fusions in particular (Lukhtanov et al. 2018). In these crosses, no evidence for assortative mating was found, and, despite the chromosome number difference between SWE and CAT, most hybrids were partially fertile (Lukhtanov et al. 2018). While some hybrid crosses have as much egg‐adult survival as pure lines, there was on average a hybrid breakdown in the F_2_–F_4_ generations, with a 42% mean egg‐to‐adult survival compared to pure lines (Lukhtanov et al. 2018). In addition, larva‐to‐adult survival was 77% compared to pure lines (Lukhtanov et al. 2018). This begs the question of whether the extensive chromosome fissions and fusions separating CAT and SWE *L. sinapis* are involved in hybrid inviability. Here, we (i) map the genomic underpinnings of hybrid inviability in *L. sinapis*, (ii) test for a direct effect of rearrangements on hybrid inviability, (iii) explore patterns consistent with an indirect effect of rearrangements on hybrid inviability by investigating the associations between recombination, chromosomal fissions and fusions, and hybrid inviability, and (iv) infer the evolution of hybrid inviability using population genomic methods.

## 2 Materials and Methods

### 2.1 Study System and Crosses

We crossed SWE and CAT *L. sinapis* and their F_1_ hybrids to establish a large set of F_2_ individuals to characterise the genetic underpinnings of hybrid inviability. The karyotypes of the parental individuals were not characterised, but we can confidently assume that they are representative of the parental populations. Some intrapopulation variation in karyotype has been observed, but the distributions of chromosome numbers for SWE and CAT are far from overlapping (Dincă et al. 2011; Höök et al. 2023; Lukhtanov et al. 2018). Two ♀SWE × ♂CAT and five ♀CAT × ♂SWE (all offspring of wild‐caught individuals) pairs were crossed in the lab in 2018 (Figure S3). F_1_ offspring were diapaused (as pupae) at 8°C in a cold room. and eight F_1_ × F_1_ crosses were performed in the spring of 2019. Mated F_1_ females were separated in individual jars where they had access to sugar water and bird's‐foot trefoil ( *Lotus corniculatus* ) for egg‐laying. Females were transferred to new jars with fresh host plants, and sugar water was replaced daily until they stopped laying eggs. A maximum of 10 of the first‐laid eggs from each female (*n* = 10 from seven females and *n* = 3 from one female; *n* = 73 in total) were sampled 3 days after laying (the “egg pool”). The developmental progress is not known for *Leptidea* at this stage, but the embryo of e.g., the Nymphalid butterfly *Pararge aegeria* is ~50% developed, has a larval‐like appearance, and is undergoing dorsal closure (Braak 2018). F_2_ offspring were reared in individual jars with *ad libitum* access to the host plant *L. corniculatus* . All jars were kept in a room that varied in temperature between 23°C and 27°C under a 16:8 h (h) light:dark regime until 28/5 2019 and a 20:4 h regime thereafter.

### 2.2 Survival Experiment

**FIGURE 1:**
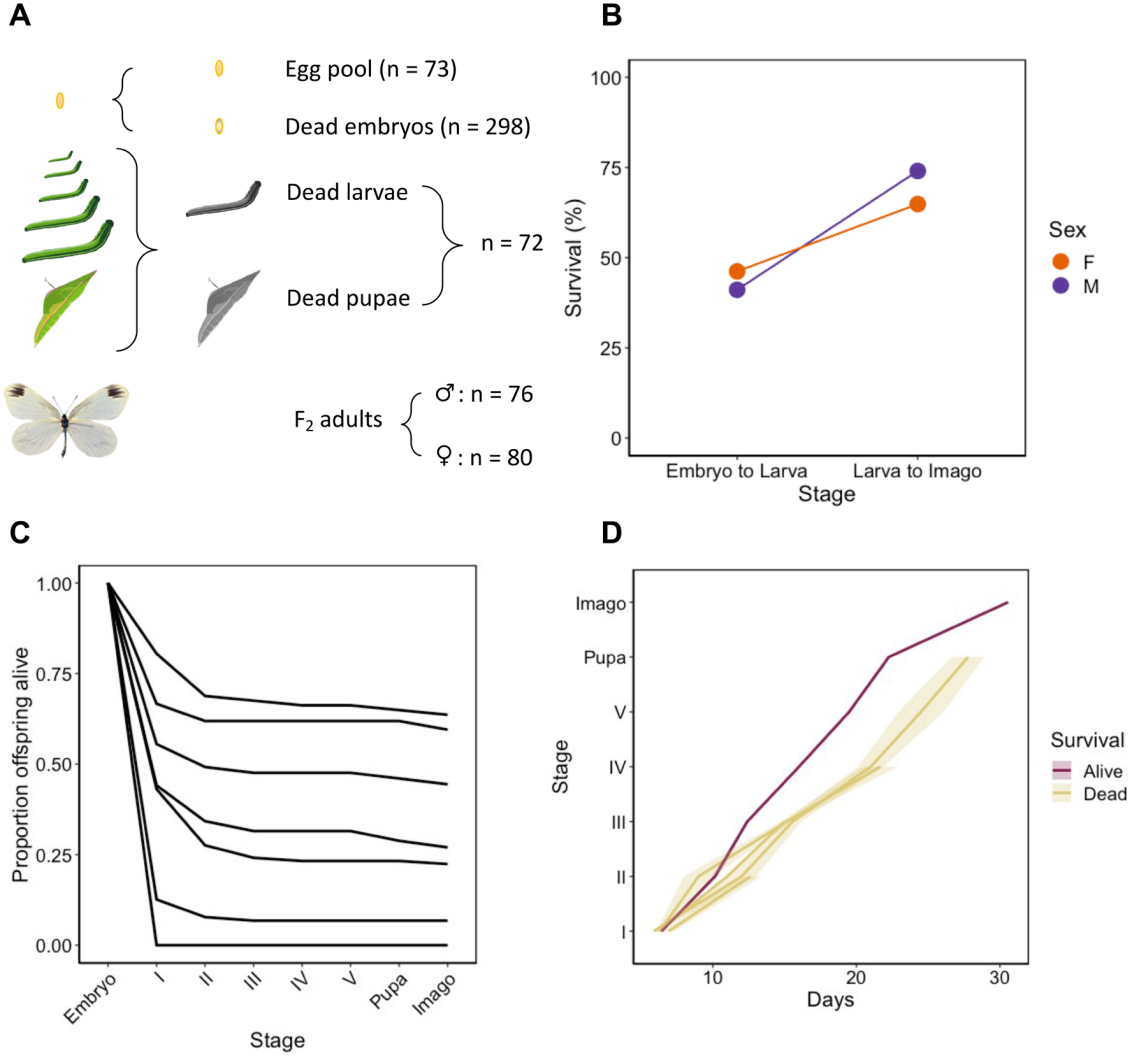
Summary of results from the F_2_ survival experiment produced by crosses between *L. sinapis* chromosomal races. We monitored cohorts of F_2_ offspring until death or emergence as adults and scored developmental stage and survival status. (A) Numbers of individuals in each pool. (B) Survival proportions across the major developmental transitions from egg to larva and from larva to imago for each sex. Overall survival was 30% for both sexes. (C) Survival curves throughout development per family. Numbers I–V represent the five larval instars. (D) Comparison of the average developmental time trajectories between alive and dead F_2_ offspring of different lifespans. Days (*X*‐axis) represent the time until reaching the corresponding stage. Shaded regions illustrate the 95% confidence intervals (narrow and hardly visible in the *Alive* group due to the large sample size of that cohort).

All egg‐laying jars were monitored daily for hatched F_2_ offspring. After hatching, Instar I larvae were separated into individual jars with access to *ad libitum* *L. corniculatus* . All individual F_2_ offspring were monitored daily, and developmental stage (and time of day) were scored until they were found dead or emerged from the pupae as imagines (adults). Each day, all offspring of a certain dam were monitored in order, but families were monitored in random order and therefore at slightly different timepoints. The variation in development time caused by this monitoring schedule was negligible (Figure 1D). Individuals that were found dead were immediately stored at −20°C. We classified embryos as dead if they had not emerged from the egg after 9 days. Emerged imagines (the “*Alive*” category) were sacrificed and stored at −20°C.

### 2.3 DNA Extractions and Sequencing of Pools

DNA was extracted using standard phenol‐chloroform extraction protocols. DNA from pupae and imagines was extracted for each individual separately, while dead larvae, dead embryos, and eggs were extracted individually or in pools of 2–21 individuals, grouped by dam (Table S1). For each sequencing pool, we used equimolar amounts of DNA adjusted by the number of individuals in each extraction pool. For example, we used twice the amount of DNA from an extraction pool with *n* = 4 compared to an extraction pool with *n* = 2. We sequenced five pools: eggs (*n* = 73), dead embryos (*n* = 298), dead larvae and dead pupae (*n* = 72), F_2_ adult females (*n* = 80), and F_2_ adult males (*n* = 76) (Table S1). Illumina TruSeq PCR‐free library preparations and whole‐genome re‐sequencing (2 × 151 bp paired‐end reads with 350 bp inserts) on one Illumina NovaSeq6000 (S4 flowcell) lane were performed by NGI, SciLifeLab, Stockholm. Sequence data is deposited at the European Nucleotide Archive under accession PRJEB69278 (Boman et al. 2023). See Table S1 for more information about DNA extraction and sequenced pools.

### 2.4 Population Resequencing Data, Variant Calling, and Inference of Fixed Difference Markers

To track the ancestry of alleles in the F_2_ offspring pools, we inferred fixed differences using individual whole‐genome population re‐sequencing data from 10 CAT and 10 SWE *L. sinapis* males, as well as two *L. juvernica* and two *L. reali* males (Talla et al. 2019). Reads < 30 bp long and with a Phred score < 30 were removed, and adapters were trimmed using TrimGalore ver. 0.6.1, a wrapper for Cutadapt ver. 3.1 (Martin 2011). Trimmed reads were mapped to the Darwin Tree of Life reference genome assembly for *L. sinapis*—a male individual from Asturias in northwestern Spain with karyotype 2*n* = 96 (Lohse et al. 2022)—using bwa *mem* ver. 0.7.17 (Li 2013). Variants were called with GATK (van der Auwera and O'Connor 2020), quality filtered with standard settings (Table S2), and used as a training set for base‐quality score recalibration (Depristo et al. 2011). Recalibrated reads were subsequently used for a second round of variant calling. Fixed differences were inferred for SNPs (i.e., excluding indels) with different alleles homozygous in all 10 CAT and SWE individuals, respectively, allowing no missing data (*n* = 27,720). These fixed differences were used as diagnostic marker loci in the PoolSeq analysis.

### 2.5 PoolSeq Read Mapping and Variant Calling

PoolSeq Illumina paired‐end reads were trimmed and adapters were removed using TrimGalore ver. 0.6.1, a wrapper for Cutadapt ver. 3.1 (Martin 2011). In addition, seven bp were trimmed from the 3′ end of all reads with a Phred score < 30. Quality‐filtered reads were aligned to two modified versions of the Asturian *L. sinapis* reference genome assembly (Lohse et al. 2022), using bwa *mem* ver. 0.7.17 (Li 2013). To reduce the impact of potential reference bias, we repainted the reference prior to mapping using either the CAT or the SWE allele for all inferred fixed differences, i.e., a “Swedenized” and “Catalanized” reference, respectively. For downstream analyses, we used the average allele frequency for both mappings to obtain a better estimate of the true allele frequency. Deduplication was performed using Picard *MarkDuplicates* ver. 2.23.4, and reads with mapping quality < 20 were removed. Allele frequencies were estimated for diagnostic loci (fixed differences between parental populations) using MAPGD *pool* (Lynch et al. 2014), which employs a likelihood ratio test to identify variants. Only markers with a likelihood ratio *p*‐value < 10^−6^ were kept for downstream analyses. For each locus, we used the allele frequency of the SWE allele in downstream analyses.

### 2.6 Quantitative Genetics Analyses

We tracked the pedigree of all F_2_ offspring in the survival experiment and performed a quantitative genetic analysis to determine the heritability for survival. Genetic variance–covariance matrices were computed using the R package Nadiv (Wolak 2012). Heritability was determined using Bayesian inference of the “animal model” as implemented in the R package mcmcGLMM (Hadfield 2010). In this framework, values of genetic variance are sampled from a prior distribution, and parameter space is explored using a Markov Chain Monte Carlo method to form a posterior distribution of genetic variance. In the first model, we used survival as the response variable and the genetic variance–covariance matrix as the random predictor to quantify the narrow‐sense heritability in survival. Since survival is a binary trait, we used a threshold link function. Both an uninformative prior (*V* = 1, *nu* = 1^−6^) and a parameter‐expanded prior (*V* = 1, *nu* = 1, *alpha.mu* = 0, *alpha.V* = 1000) were applied. Both prior settings resulted in an estimated heritability within one percentage point of each other, indicating a low influence of the prior settings on the posterior distribution (Tables S3 and S4; Figure S4). To calculate the heritability on the observed data scale, we used *model = binom1.probit* in the R package QGglmm (de Villemereuil et al. 2016). For models with development time as a Gaussian response variable, we used a random slopes model (*random = ~us(1 + Stage):animal + animal*) with *Sex* + *Survival* as fixed effects. We used both parameter‐expanded and uninformative priors, and both settings gave qualitatively similar results (Tables S5 and S6).

### 2.7 Inference of Candidate Regions

First, we used a Kolmogorov–Smirnov (KS) test on weighted allele frequencies between the *Alive* (adult males and females) and *Dead* (dead embryos and dead larvae + dead pupae) groups of pools, following Lima and Willett (2018), to detect chromosomes with a significantly different distribution of allele frequencies. This test was performed for marker loci on individual chromosomes separately, and we used a Bonferroni correction to correct for multiple testing. The null hypothesis in the KS test is that both *Alive* and *Dead* follow the same distribution of allele frequencies. Candidate regions for hybrid inviability were identified by calculating allele frequency differences between the *Alive* and the *Dead* groups of pools, weighted by sample size for each pool (see Table S1). We used all 27,240 markers with data for all sequence pools × mapping combinations (i.e., 4 × 2). A generalised additive model (GAM), using cubic splines as smoothener from the *R* package mgcv, as implemented in the *R* package ggplot2 *geom_smooth* function, was used to get allele frequency trajectories along each chromosome (Wickham 2016; Wood 2011). We used the GAM cubic spline approach since it fitted the observed distribution of marker loci well without overfitting small regions of chromosomes with deviating allele frequencies. Candidate regions were defined as regions where the 95% confidence interval exceeded an absolute allele frequency difference cutoff (in general 0.05, but we also applied stricter cutoffs for comparison; see below). The allele frequency differences between genomic regions are expected to be small on average, since most haplotypes are expected to be fit for a typical recessive two‐locus incompatibility (Fitzpatrick 2008; Lima and Willett 2018; Orr 1995; Text S1). We performed simulations to determine the false positive rate when using the GAM analysis on the *Alive* vs. *Dead* comparison (see Text S1 for more details). We also used an alternative and more stringent method to define candidate regions by performing a bulk‐segregant QTL‐seq analysis using the R package QTLseqr (Mansfeld and Grumet 2018). All QTLs with an allele frequency difference greater than the 95% CI compared to simulated data and which included more than one SNP were retained as candidate loci. This represented a mean cutoff level of 0.14 (i.e., the observed mean allele frequency difference between pools). Note that this cutoff is based on the mean of the smoothened values obtained from QTL‐seq, and it is therefore not directly comparable to the CI‐based cutoff applied for the generalised additive model. It should also be noted that there are several important caveats with the QTL‐seq analysis. The implementation of the model assumes equal sample sizes of pools (e.g., dead larvae + dead pupae and dead embryos are given equal weight despite the approximately 4‐fold sample size difference) and therefore does not take into account multiple sequence pools (such as our *Alive* and *Dead* groups of pools). In addition, the CIs simulated by QTLseqr are ignorant of the degree of linkage between marker loci and are thus incorrect. Taken together, we expected QTL‐seq to be overly conservative with our experimental design.

### 2.8 Mapping Chromosomal Rearrangements to the Reference Assembly

To map chromosomal rearrangements to the Asturian *L. sinapis* genome assembly, we performed pairwise LASTZ ver. 1.04 (Harris 2007) whole‐genome alignments to previously published reference assemblies for a SWE and a CAT male, respectively (Höök et al. 2023). Parameters used for both runs of LASTZ were *M = 254*, *K = 4500*, *L = 3000*, *Y = 15,000*, *C = 2*, *T = 2*, and *–matchcount = 10,000*. See Figure S1 for a synteny map of the alignment. We used previously available data on the polarisation of fission and fusion events, which were based on synteny analysis of four chromosome‐level genome assemblies: one each of SWE and CAT *L. sinapis*, as well as the outgroup species *L. reali*, and *L. juvernica* (Höök et al. 2023; Näsvall et al. 2023). For example, if a chromosome is fused in *L. juvernica*, *L. reali* and SWE *L. sinapis* but unfused in CAT *L. sinapis*, then the rearrangement was inferred to be a derived fission in the CAT lineage (Fission CAT; see Figure S2 for a graphical example). Chromosomes that had a shared breakpoint with outgroups *L. juvernica* and *L. reali* were classified as having unknown polarisation (Höök et al. 2023; Näsvall et al. 2023). The sample size of each rearrangement type was Fusion SWE = 5, Fission CAT = 5, and Unknown polarisation = 4. These three categories all share the property that SWE has the fused state (one chromosome) and CAT has the unfused state (two chromosomes). We therefore refer to these as simple rearrangements. For simple rearrangements, we also defined evolutionary breakpoint regions (EBRs) as regions ± 0.5, 1, 1.5, 2, and 3 Mb from the fusion/fission point. For readability, we focus on ± 1 Mb regions in the main text unless otherwise stated. For reference, we used (putatively) syntenic chromosomes between populations (*n* = 5). We also analysed 20 putative chromosomal inversions and 6 chromosomes associated with more complex rearrangements (chain rearrangements).

### 2.9 Recombination Rate Estimates

Recombination rate estimates were obtained from pedigree‐based linkage maps from the Swedish and the Catalan populations (for details see refs: Höök et al. 2023; Näsvall et al. 2023). The genetic distance for each within‐chromosome marker pair was divided by the physical distance to calculate the expected number of crossover pairs per megabase pair (centiMorgans/Mb).

### 2.10 Demographic Inference

To infer the demographic history of the SWE and CAT populations of *L. sinapis*, we used the previously described population re‐sequencing data. For this analysis, SNPs were filtered to obtain the most reliable variants (Table S2). The resulting SNP data set was thinned using vcftools ver. 0.1.16 (Danecek et al. 2011) to ensure that SNPs were at least 10 kb apart. This decreases the impact of physical linkage between sites while ensuring that the whole genome is represented. As a final filtering step, we removed all remaining SNPs inside coding sequences to reduce the impact of selection on the demographic inference. The final SNP set consisted of 59,823 variants. We computed the joint minor allele frequency (MAF) spectrum using easySFS (Overcast 2022). The parameters in the demographic model were inferred using GADMA ver. 2 (Noskova et al. 2023), which employs a genetic algorithm to optimise parameter values. As an engine in the inference, we used Moments (Jouganous et al. 2017), which fits the observed joint MAF spectrum to simulated data using ordinary differential equations. To transform relative values into estimates of *N*_e_ and time in generations since divergence, we assumed a callable sequence length of 7,489,125 bp after filtering, based on *π* = 0.008 (note that this is lower than the observed levels of genetic diversity due to population expansions). The mutation rate was set to 2.9 × 10^−9^ per base pair and generation—an estimate from a pedigree‐based analysis in *Heliconius melpomene* (Keightley et al. 2015). Two demographic models were inferred (Isolation and Isolation‐with‐migration) and compared using the Akaike information criterion. Confidence intervals for demographic parameters were estimated based on 100 bootstrap replicates of the joint MAF using the Godambe information criterion (Coffman et al. 2016).

### 2.11 Population Genetic Analyses

We filtered the population resequencing all‐sites variant call format file (including variant and invariant sites) based on depth by marking individuals with < 5 and > 25 reads as missing data using BCFtools *filter* (Danecek et al. 2021). Population genetic summary statistics (*F*_ST_, *D*_*XY*_, and *π*) were estimated using *pixy* (Korunes and Samuk 2021). We used Hudson's estimator of *F*_ST_, as recommended by Bhatia et al. (2013). All population genetic summary statistics were estimated in 10 kb genomic windows for three sets of windows: genome‐wide (all windows), hybrid inviability candidate regions, and non‐candidate regions. We used ANOVA with a linear model (*X ~ Chromosome + Type*, where *Type* signifies candidate and non‐candidate regions and *X* represents *F*_ST_, *D*_*XY*_, and *π*_SWE_ and *π*_CAT_, respectively) to determine whether the population genetic summary statistics varied with the type of genomic region while controlling for chromosomal effects such as faster differentiation on the Z chromosomes.

### 2.12 Genomic Resampling Methods

We used a resampling method to evaluate the association between hybrid inviability candidate regions and other sets of genomic features using a custom script. Chromosomes were randomly chosen, weighted by their length. Coordinates within chromosomes were sampled according to the length of the testing set X, and the overlap between testing set X and reference set Y was calculated. We calculated a two‐tailed empirical *p*‐value as 2*r*/*n* for *r/n* ≤ 0.5 and 2 (1 − *r/n*) for *r/n* > 0.5, where *r* is the number of replicates with an overlap greater than or equal to the overlap for the observed data. Enrichment was defined as the following odds ratio for the two sets (X and Y):

$$\text{Odds ratio}=\frac{\text{Overlap between}\;X\;\text{and}\;Y}{\text{Total length of}\;X}/\frac{\text{Total length of}\;Y}{\text{Genome length}}.$$

## 3 Results

### 3.1 Equal Survival of Males and Females

We crossed CAT (2*n* = 106–108) and SWE (2*n* = 57, 58) chromosomal races of *L. sinapis*. Only males successfully eclosed after diapause in the ♀SWE × ♂CAT (*n* = 2) crosses, an effect that could constitute a diapause‐associated sex‐specific F_1_ inviability, but a larger sample size is needed to exclude chance. Both males and females eclosed in the ♀CAT × ♂SWE (*n* = 5) crosses. We further crossed eight F_1_ females with five F_1_ males. F_1_ females (♀CAT × ♂SWE) laid 3–126 eggs, producing 615 F_2_ offspring in total (Figure S3) The first ≤ 10 offspring of each female were collected to form a random pool of eggs, following Lima and Willett (2018). We performed a hybrid survival experiment by monitoring the development of the remaining F_2_ offspring and observed an overall survival of 30% for both males (30.28%, 95% confidence interval: [30.23–30.32]) and females (30.11% [30.07–30.15]; Figure 1B). Most F_2_ offspring died prior to hatching from the egg (“dead embryos,” 44% survival), and the proportion of offspring surviving until the imago stage varied widely among families (Figure 1B,C). Since survival could be due to both genetic and environmental effects, we performed quantitative genetic analyses to estimate the genetic component of this trait. We observed a 38% narrow‐sense heritability for survival (Tables S3 and S4; Figure S4). We also found that individuals that died during the larval or pupal stages had slower developmental rates (Random slopes model; *p* ≈ 0.002; Figure 1D; Tables S5 and S6).

### 3.2 Genomic Architecture of F_2_ Hybrid Inviability

**FIGURE 2:**
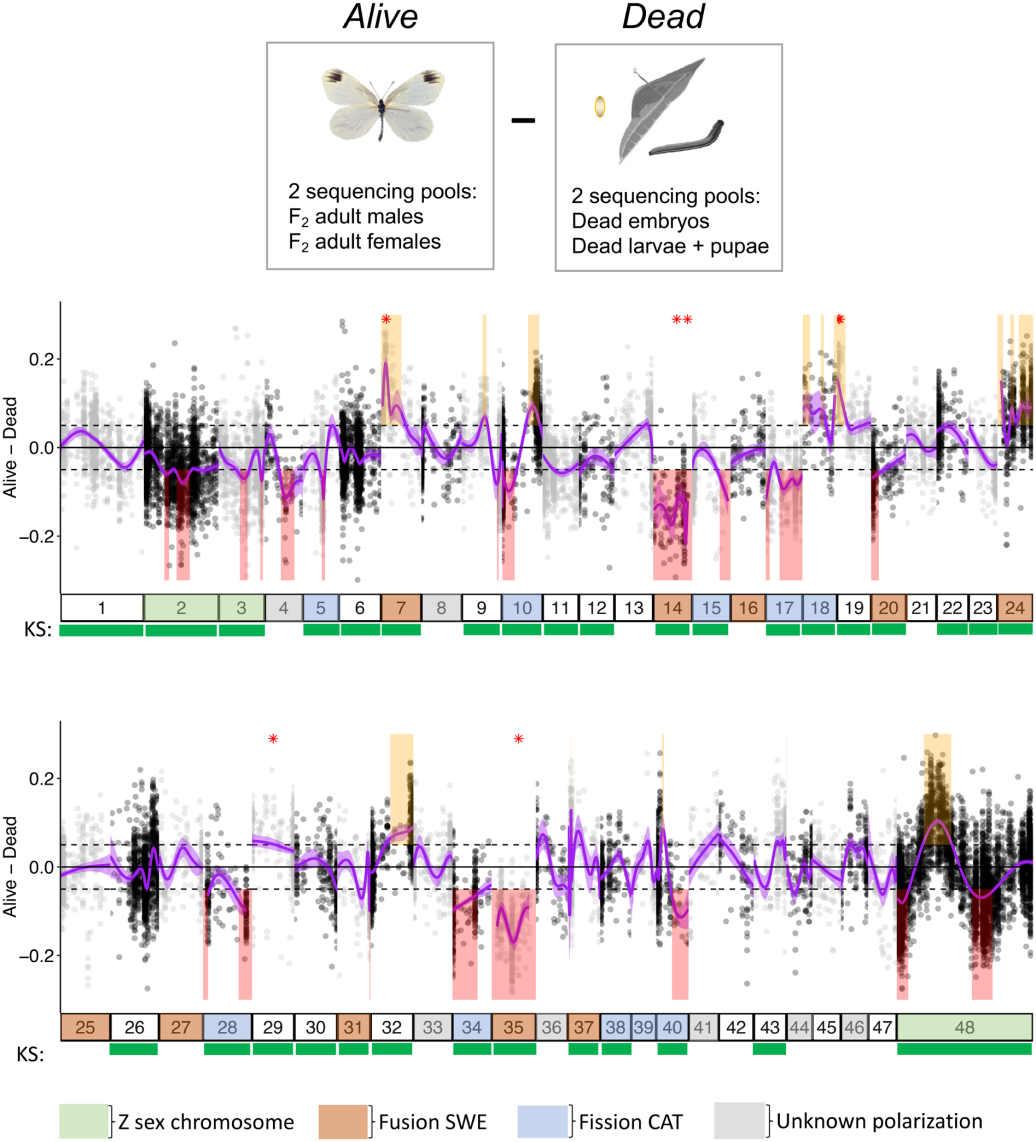
Genomic architecture of F_2_ hybrid inviability in *L. sinapis* mapped by comparing allele frequencies of the *Alive* and *Dead* groups of pools. Y‐axes represent the allele frequency difference between the pools *Alive* (F_2_ adult males and females) and *Dead* (dead embryos and dead larvae + pupae). X‐axes show the chromosomes (numbered bars) ordered by size, except chromosome 48, which is the ancestral Z_1_ chromosome of Lepidoptera. Dots show the position and SWE allele frequency of the 27,240 markers. The purple curve represents a generalised additive model (cubic splines) fitted to the allele frequency difference between pools. Shaded areas in the graph represent regions where the *Alive* pool had an excess of SWE (yellow) or CAT (red) alleles, respectively (i.e., where the 95% CI of the curve > |0.05|). Beneath chromosomes are shown the results from the Kolmogorov–Smirnov (KS) test, with a green bar representing a significant difference between *Alive* and *Dead* for a chromosome. Red asterisks (*) indicate the mid position of candidate regions identified using QTLseqr (Figure S5). The colours of chromosomes indicate if they represent Z sex chromosomes (green),  derived fusions in the SWE population (brown), derived fissions in the CAT population (blue), or segregating fission/fusion polymorphisms with unknown polarisation (grey). Note that only simple rearrangements (involving two unfused elements) are shown.

To study hybrid inviability while simultaneously excluding other postzygotic barriers, we sequenced several experimental pools and compared allele frequencies between F_2_ individuals surviving to adult stage (*Alive*, two sequencing pools) and individuals that died during development (*Dead*, two sequencing pools; Figure 1A; Table S1). We compared the allele frequencies at marker loci between the *Alive* and *Dead* groups of pools to identify regions potentially associated with hybrid incompatibilities (candidate regions). Using the KS test, we observed significant differences in allele frequency distributions between *Alive* and *Dead* in 32 out of 48 chromosomes (Figure 2; Table S7). Using the GAM analysis, we found 37 candidate regions associated with hybrid inviability spread over 21 chromosomes with significantly deviating allele frequencies compared to random expectations. Since the GAM analysis is a novel approach, we used simulations based on our experimental setup and determined a false positive rate of 2.3%–10.8% using a 0.05 allele frequency difference cutoff in the *Alive* vs. *Dead* comparison (see Text S1 for details). Hence, the absolute majority of identified candidate regions should represent true positives. In the *Alive* group, 22 regions had an excess of the CAT and 15 had an excess of the SWE variant, respectively (Figure 2). Regions with an excess of the CAT variant comprised 13.2% (90.5 Mb) of the genome in the *Alive* group, while regions with SWE ancestry comprised 6.6% (45.1 Mb). Candidate regions varied in size from 73 to 14.9 Mb and sometimes spanned entire chromosomes, such as chromosomes 14 and 35, which are fused in the SWE population (Figure 2). Importantly, the KS‐test and the GAM analysis identified the same chromosomes as significantly different between *Alive* and *Dead* in many cases (correlation coefficient = 0.53, *p* ≈ 9 × 10^−5^), with the latter approach being more conservative (Table S7). As a stringent complementary method to detect significant allele frequency shifts between pools, we used the QTLseqr method on the *Alive* vs. *Dead* data set (Figure 2; Figure S5). All (*n* = 7) except one of the QTLs detected in this analysis were located inside five of the 37 candidate regions identified in the GAM analysis. We classified these seven regions as large‐effect loci since they were located in genomic regions with especially pronounced allele frequency differences (Figure 2).

We compared allele frequencies between the *Alive* and the egg pool to test whether the candidate regions detected in the *Alive* vs. *Dead* comparison could be confirmed using an alternative approach. Note that this is not a strict test of repeatability, given that the *Alive* pool was used in both analyses. We found that candidate regions in these comparisons overlapped 1.41‐fold over the random expectation (Monte Carlo *p* = 0.022, *n* = 1000). We repeated this analysis using a more stringent (0.075) frequency difference threshold for the *Alive* vs. egg pool comparison and found similar results (odds ratio ≈ 1.80, *p* = 0.014; Figure S6). The comparison between the *Alive* vs. *Dead* and the *Alive* vs. egg pools was complicated by the observation that the egg pool consisted of approximately 68% females, according to the observed read coverage on Z_1_ and Z_2_ sex chromosomes, while the *Alive* and *Dead* combination of pools had equal sex ratios (Table S1). We expect that sex ratios of pools, within and between comparisons, affect the predicted candidate regions since the Z_1_ and Z_2_ chromosomes are hemizygous in females, which will affect the expression of incompatibilities caused by recessive variants. Consequently, trying to detect developmental stage‐specific incompatibilities by contrasting dead larvae and dead embryos would be confounded by the difference in sex ratios between these pools (Table S1).

### 3.3 Rearrangements and Hybrid Incompatibilities

Since chromosomal rearrangements might affect hybrid fitness, we investigated whether chromosomes involved in fission/fusion differences between the SWE and the CAT populations were enriched in hybrid inviability candidate regions. Enrichment analysis was performed both for the entire chromosomes involved in rearrangements in general and for the evolutionary breakpoint regions (EBRs; ± 1 Mb of an inferred fission/fusion breakpoint) more specifically (see Figure S2 for a visual representation of EBRs). For the entire chromosomes, we found no significant enrichments of candidate regions after correcting for multiple testing (Table 1). In the EBRs, however, derived fusions were significantly enriched for candidate regions (odds ratio ≈ 2.08, Monte Carlo *p =* 0.006, *n* = 1000). We also tested the seven large‐effect loci identified using the QTL‐seq analysis. Four out of seven of these loci map to Fusion SWE chromosomes (odds ratio ≈ 4.87, *p* < 0.008), but none to fusion EBRs (*p* = 1; Table S8).

**TABLE 1:** Associations between chromosomal rearrangements and hybrid inviability candidate regions. Enrichment analysis was performed using genomic resampling (see Section 2 for details). Independent analysis was performed for entire chromosomes, evolutionary breakpoint regions (EBRs), and non‐EBR ends of rearranged chromosomes, respectively (Figure S2). Chromosomes with unknown polarisation are those with fission/fusion polymorphisms that are known to segregate in different *Leptidea* species. For full chromosomes and chromosome ends we also analysed syntenic chromosomes.

Category	Polarisation	Odds ratio	*p*	Adjusted *p*‐value^a^
Chromosome	Fission CAT	1.800	0.056	0.224
Chromosome	Fusion SWE	1.755	0.060	0.240
Chromosome	Unknown pol	0.324	0.240	0.960
Chromosome	Syntenic	0.176	0.080	0.320
EBR	Fission CAT	1.010	0.146	0.438
EBR	Fusion SWE	2.075	< 0.001	< 0.001
EBR	Unknown pol.	1.263	0.348	1
Non‐EBR ends	Fission CAT	2.728	< 0.001	< 0.001
Non‐EBR ends	Fusion SWE	2.021	< 0.001	< 0.001
Non‐EBR ends	Unknown pol.	0	0.435	1
Chromosome ends	Syntenic	0.505	0.774	1

^a^ Corrected for multiple testing using the Bonferroni method for each category separately.

The association between fusion EBRs and candidate regions could be due to an association between chromosome ends and hybrid inviability, rather than the fusion event itself. Consequently, we also investigated non‐EBR ends of rearranged chromosomes and ends of syntenic chromosomes. This analysis showed that there was an enrichment of candidate regions at non‐EBR ends of derived fusions (odds ratio ≈ 2.02, *p* < 0.001) and fissions (odds ratio ≈ 2.73, *p* < 0.001). Chromosomes with unknown polarisation, however, contained no candidate regions at non‐EBR ends (*p* = 1), and ends of syntenic chromosomes showed no significant enrichment (*p* = 1). Thus, candidate regions are not enriched at all types of chromosome ends, suggesting that rearrangement type is important. We also analysed EBRs and non‐EBR chromosome ends of sizes ± 0.5, 1.5, 2, and 3 Mb and got qualitatively similar results to the analysis of EBRs ± 1 Mb, showing that our conclusions are not affected by the choice of window size (Figure S7).

To further assess if the association between candidate regions and EBRs could be a consequence of differences in gene density between conserved and rearranged regions, we investigated the relationship between coding sequence (CDS) density and the candidate regions. This analysis unveiled a small but significant excess (odds ratio ≈ 1.08, *p* = 0.036) of CDS regions in candidate regions compared to the genome‐wide level. Derived fusion EBRs had a significantly lower density of CDS regions compared to the genome‐wide level (odds ratio ≈ 0.58, *p* = 0.002). Thus, CDS density cannot explain the association between candidate regions and fusion EBRs.

So far, we have only considered simple rearrangements involving two unfused elements. We also investigated two other rearrangement types: complex rearrangements involving several chromosomes, as well as inversions, but neither were significantly enriched for hybrid inviability factors (Text S2). We therefore only focus on the simple fission/fusion rearrangements in the following analyses.

### 3.4 Systematic Aneuploidy Does Not Explain Observed Hybrid Inviability at Fusions

**FIGURE 3:**
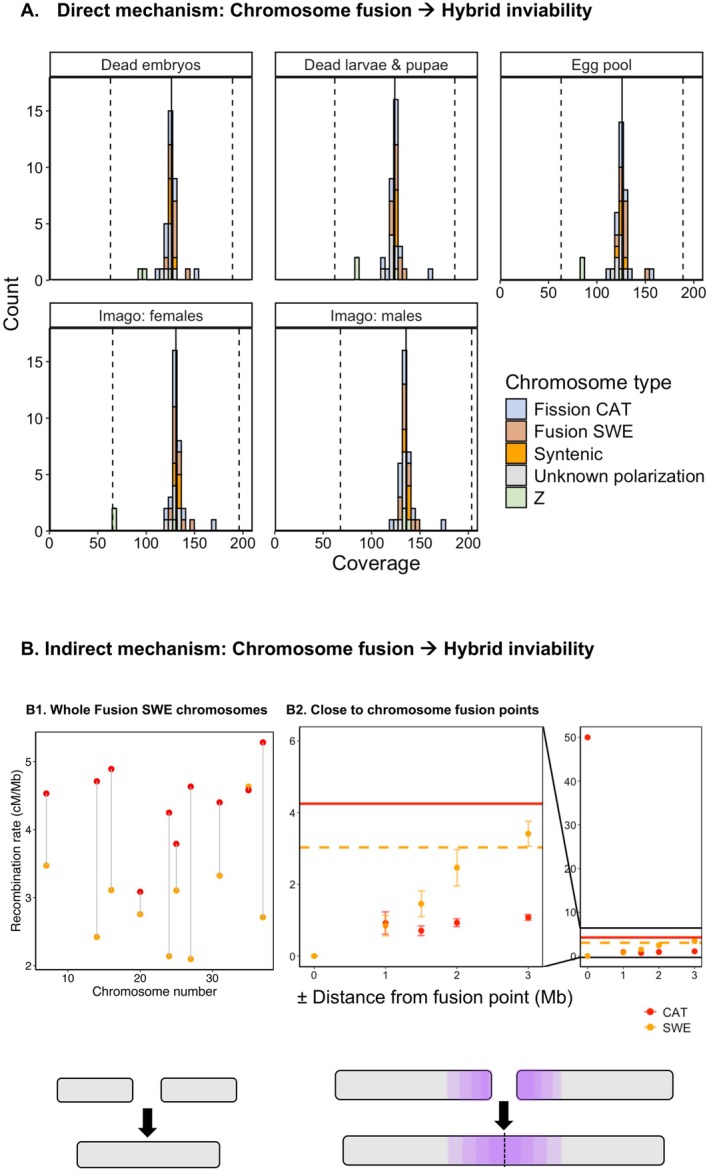
Testing aspects of the direct (A) and indirect (B) models for the relationship between chromosome fusions and hybrid inviability. (A) Sequencing coverage at fixed differences for all sequencing pools. Filled vertical lines represent the average coverage per chromosome. Dashed vertical lines from left to right represent expected coverage for systematic monosomy (e.g., two of three Z sex chromosomes in the imago female pool) and trisomy, respectively. (B1) Parental population recombination rates at Fusion SWE chromosomes in the *L. sinapis* reference assembly. (B2) Parental population recombination rates in ± 0, 1, 1.5, 2, and 3 Mb windows adjacent to chromosome fusion points, i.e., evolutionary breakpoint regions (EBRs). For ± 1–3 Mb, we show estimates of within‐chromosome recombination rates, while for ± 0, we show the effects of two loci immediately adjacent in the fused state and segregating on different chromosomes in the unfused state. Error bars represent the standard error of the mean. Solid and dashed lines show the recombination rates in the CAT and the SWE populations, respectively. Horizontal lines represent mean genome‐wide recombination rates for the CAT (red) and SWE (orange) populations.

Chromosome fission/fusion polymorphisms can lead to non‐disjunction during meiosis and the formation of aneuploid gametes (reviewed in King 1993). In frog hybrids, aneuploid gametes can survive many rounds of cell division (Gibeaux et al. 2018). It is possible that similar processes could occur in *Leptidea* hybrids, since univalents have been observed in F_1_ hybrid males (Lukhtanov et al. 2018). Investigating aneuploidy can therefore inform about the mechanism(s) relating chromosome fusions and hybrid inviability. We expect to see systematic aneuploidy if non‐disjunction during meiosis due to fusions underlies the hybrid inviability candidate regions we identified. If there is no systematic aneuploidy, we expect the relationship between fusions and hybrid inviability to be indirect, and, in that case, hybrid inviability is more likely a consequence of linked genic incompatibility factors. We investigated whether any whole‐chromosome systematic aneuploidies were present in all sequencing pools by comparing read coverage among chromosomes at marker loci. In for example, the dead embryo pool, the autosome with the highest coverage had 37% higher coverage than the average level among all autosomes (Figure 3A). In the case of aneuploidy, we would expect single autosomes to have either 50% higher coverage (trisomy), half the coverage (monosomy), or no coverage at all (nullisomy), compared to other autosomes (dashed vertical lines in Figure 3A). For surviving F_2_ males and females, the difference between the highest covered autosome and the average was 27% and 35%, respectively (Figure 3A). For both dead embryos and imagines, chromosomes 17 and 21 had the highest coverage. Neither of these two chromosomes is associated with simple derived fusions (see Figure 2). We also performed ANOVA and confirmed that significant differences between chromosome types and pools are only found when including Z chromosomes (Table S9). This indicates that it is unlikely that systematic aneuploidies are present in any pool and that the relationship between hybrid inviability and fusions that we observed is caused by other factors. In other words, while we cannot exclude that aneuploidies are causing some hybrid deaths, aneuploidies do not explain the relationship between hybrid inviability and chromosome fusions that we observed since that would require a systematic excess of one of the parental chromosomes. Therefore, we further examined an indirect mechanism of chromosomal speciation by investigating the relationships between hybrid inviability, chromosome fusions, and the recombination rate.

### 3.5 Hybrid Inviability Candidate Regions Are Characterised by Low Recombination Rates

We expected hybrid inviability candidate regions to show a reduced recombination rate compared to other parts of the genome based on several different models (see Section 1). To test this prediction, we bootstrapped genomic regions of the same sizes as the observed candidate regions and extracted recombination rates in those regions from population‐specific linkage maps (Figure S8). Importantly, since the underlying regional recombination rate variation can affect the size distribution of candidate regions, we calculated the arithmetic mean recombination rate without normalising for sequence length. We found that the recombination rates in candidate regions in both the SWE (2.39 cM/Mb; Monte Carlo *p* ≈ 0.027, *n* = 100,000) and the CAT population (3.42 cM/Mb; *p* ≈ 0.026) were significantly lower compared to the genome‐wide rates (3.03 and 4.25 cM/Mb for SWE and CAT, respectively).

Low recombination rates could explain part of the association between hybrid inviability and chromosome fusions. We found a significantly lower recombination rate for the fused (SWE; mean ≈ 2.98 cM/Mb) compared to the unfused (CAT; mean ≈ 4.15 cM/Mb) state at Fusion SWE chromosomes (two‐sample paired Wilcoxon tests; *p* ≈ 0.004, Figure 3B). When a chromosome fusion occurs, loci in the vicinity of the fusion point that were segregating independently become tightly linked. The reduced recombination rate near fusions is expected to extend over a larger region since the central parts of large chromosomes have a reduced recombination rate compared to the genomic average rate in butterflies (Davey et al. 2017; Näsvall et al. 2023; Palahí et al. 2023; Shipilina et al. 2022). In line with this, we found that derived fusion EBR regions (± 1 Mb of an inferred breakpoint), had significantly reduced recombination rates compared to the genome‐wide rate in the SWE (fused state) population (one‐sample Wilcoxon tests; *p* < 0.05; Figure 3D; Table S10). In addition, the CAT (unfused state) also had lower recombination rates (*p* < 0.05; Figure 3B; Table S10), in line with reduced recombination at chromosome ends in Lepidoptera (Davey et al. 2017; Näsvall et al. 2023; Palahí et al. 2023; Shipilina et al. 2022). Nevertheless, in the immediate vicinity of the fusion point, unfused chromosomes segregate independently (i.e., free recombination), and fused chromosomes have essentially zero recombination (Figure 3B). EBR and non‐EBR ends of fusions did not have significantly different recombination rates (two‐sample Wilcoxon tests; *p* > 0.05; Figure S9). We also investigated the derived fissions and fission/fusion rearrangements with unknown polarisation and found significantly lower recombination rates at non‐EBR ends compared to EBRs for both (*p* < 0.005; Figure S9; Table S11). In conclusion, fusions—but not other types of rearrangements—had reduced recombination rates in the EBRs.

### 3.6 Higher Levels of Genetic Differentiation in Hybrid Inviability Candidate Regions

**FIGURE 4:**
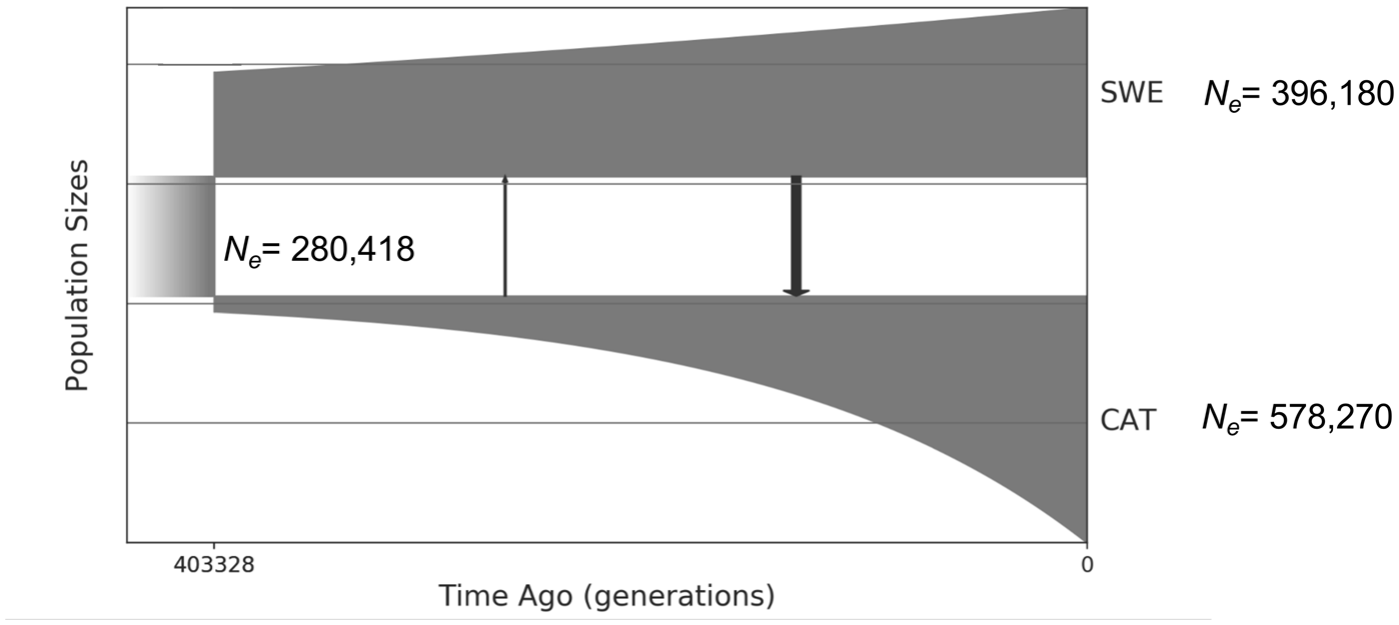
Demographic history of SWE and CAT populations. Sizes of boxes represent the effective population size (*N*_e_). The current (at *T* = 0) effective population sizes were estimated to be 578,270 [196,500–954,426] for CAT and 396,180 [112,391–674,349] for SWE. Arrows connecting boxes are directional migration rates averaged across the entire epoch. Arrow widths are scaled to illustrate the intensity of migration. See Table S12 for more information on parameter estimates and Figure S10 for model fit diagnostics.

To further understand the evolutionary origins of hybrid inviability, we investigated the demographic history and genomic landscape of differentiation and divergence using population resequencing data from 10 males each of CAT and SWE *L. sinapis*. First, we investigated the demographic history of the populations using GADMA (Figure 4). Models incorporating continuous gene flow provided a superior fit to the observed joint minor allele frequency spectrum compared to models without migration (d_AIC_ = 3715; Figure 4; Figure S10; Table S12). Inferred gene flow was higher from the SWE to the CAT population (M_SWE➔CAT_ = 1.07 migrants per generation, 95% CI: 0.5–1.6) than vice versa (M_CAT➔SWE_ = 0.18 migrants per generation, 95% CI: 0–0.4; Figure 4 and see Table S12 for inferred parameter values). We contrasted population genetic summary statistics in candidate regions with the rest of the genome to get a better understanding of the processes that may have influenced their evolution. On average, *F*_ST_ between parental samples (CAT and SWE), measured in 10 kb windows, was significantly higher in candidate regions (0.27) compared to non‐candidate regions (0.26, ANOVA, *p* < 0.001; Figure S11; Table 2; Table S13). Conversely, *π*_CAT_ was significantly lower in candidate regions than in non‐candidate regions (*p* < 0.001). We found no significant differences in *π*_SWE_ and *D*_*XY*_ in candidate regions compared to the rest of the genome when controlling for between‐chromosome variation (*p* > 0.05; Table S13).

**TABLE 2:** Population genetics of hybrid inviability candidate regions. Estimated average [95% confidence intervals] population genetic summary statistics in non‐overlapping 10 kb windows for candidate and non‐candidate incompatibility regions in the genome, respectively. *p*‐values were obtained through ANOVA; see Table S13 for more details.

Statistic	Candidate regions	Non‐candidate regions	*p*
*F*_ST_	0.2725 [0.2701–0.2749]	0.2602 [0.2578–0.2628]	< 0.001
*D*_*XY*_	0.0115 [0.0114–0.0116]	0.0122 [0.0121–0.0122]	0.256
*π*_SWE_	0.0081 [0.0081–0.0082]	0.0086 [0.0086–0.0087]	0.79
*π*_CAT_	0.0087 [0.0087–0.0088]	0.0094 [0.0093–0.0094]	< 0.001

*Note: F*_ST_, *D*_*XY*_, and *π* were rounded to four decimals.

## 4 Discussion

Here, we used a combination of approaches to investigate the genetic underpinnings of hybrid inviability between two populations of wood whites that differ in karyotype structure due to a large number of chromosomal rearrangements. Our detailed characterisation of survival in hybrid offspring, the mapping of candidate hybrid inviability loci, and investigations of the differences in population genomic signatures at candidate loci compared to the genome in general revealed that the evolution of hybrid inviability is associated with the extensive chromosomal rearrangements that have occurred in different lineages of *L. sinapis*.

### 4.1 The Genetic Basis of Hybrid Inviability—Comparisons of Dead and Alive Offspring

Here we added a novel twist to the PoolSeq approach of mapping hybrid inviability (Lima and Willett 2018) by sampling both surviving and dead F_2_ offspring from crosses between parental lineages with distinct karyotypes. Using the *Alive* vs. *Dead* approach, instead of comparing to the Mendelian expectations, allowed us to distinguish hybrid inviability from earlier acting causes of segregation distortion, such as hybrid sterility and meiotic drive (Boman et al. 2024). It is important to emphasise that the identified loci are no more than candidate regions, and further experiments would be necessary to identify the genes and specific genetic differences involved, as well as the potential epistatic interactions that cause hybrid inviability. Identifying genome‐wide epistatic interactions requires genotypes from recombinant offspring, huge sample sizes, and data from many generations of intercrossing. This is the reason why it has only been done indirectly or on a small scale, far from capturing all the expected epistatic interactions (Li, Schumer, and Bank 2022; Satokangas et al. 2020; Szabo and Cutter 2024). Nevertheless, identifying genomic regions associated with hybrid incompatibilities (as we do in this study), has proven fruitful for our understanding of how postzygotic isolation evolves (Coyne and Orr 2004; Maheshwari and Barbash 2011).

We further outline two of the challenges with the PoolSeq method to study hybrid incompatibilities below. First, since we expect recessive hybrid incompatibilities to be at play in a system with F_2_ hybrid breakdown, such as in *L. sinapis*, each mating will generate a relatively high proportion of viable offspring, and allele frequency deviations from the expected value of 0.5 will in most cases be modest (Lima and Willett 2018). This reduces the power to detect loci associated with inviability. Here we compared allele frequencies between pools of both *Alive* and *Dead* F_2_ offspring, which increases the power to some extent (Text S1). This is because alleles enriched for the variant with SWE ancestry in the *Alive* pool necessarily will be enriched for the variant with CAT ancestry in the *Dead* pool, and vice versa (Text S1). Second, if hybrid inviability is caused by a combination of a few loci with large effects and many small‐effect loci (i.e., the trait is polygenic), as has been observed for example, for hybrid sterility in the *Drosophila simulans* clade (Presgraves and Meiklejohn 2021) and in *Papilio* butterfly hybrids (Xiong et al. 2023), the power to detect the true genomic architecture in an F_2_ cross with a limited number of recombination events will be low. For the present study, it means that we cannot exclude that there are many more loci with small effects involved in hybrid inviability. We did, however detect a set of candidate regions with sufficiently large effect sizes, which allowed for further investigation of the evolution of hybrid inviability. In particular, we looked for significant enrichment between hybrid inviability candidate regions and chromosomal rearrangements. Sometimes the association between rearrangements and phenotypes is investigated without testing for enrichment (Wellband et al. 2019), which is not necessarily an invalid approach. Importantly, when finding overlaps and testing for an enrichment but seeing no signal, we still cannot exclude that a certain rearrangement type might be important for the evolution of a phenotype. However, observing a significant enrichment should increase our confidence in the importance of a certain rearrangement type for the genetic architecture of a trait, in our case hybrid inviability. Of all the rearrangement types we tested, chromosome fusions were most clearly associated with hybrid inviability. We thus focus on the relationship between hybrid inviability and chromosome fusions in the following discussion.

### 4.2 Evolution of Hybrid Inviability Through Association With Chromosome Fusions

There are several possible reasons for an association between hybrid inviability and chromosomal rearrangements. A genetic correlation between traits can be caused by pleiotropy, tight physical linkage between independent genes affecting the traits, or both. In the case of hybrid inviability and chromosome fusions, a pleiotropic mechanism would be that hybrid inviability is caused directly by the changed chromosome structure itself. A physical linkage explanation would instead be that the fixation of a chromosomal rearrangement leads to the fixation of linked genic incompatibility factors. Both pleiotropy and physical linkage could cooccur, as has been inferred from F_2_ crosses between populations of the Australian grasshopper *Caledia captiva*, which differ in both karyotype and fixed genetic differences (Shaw, Coates, and Wilkinson 1986). Since we did not observe any systematic aneuploidies for rearranged chromosomes, our data supports (but does not prove) the physical linkage model, i.e., an indirect relationship between chromosome fusions and hybrid inviability.

**FIGURE 5:**
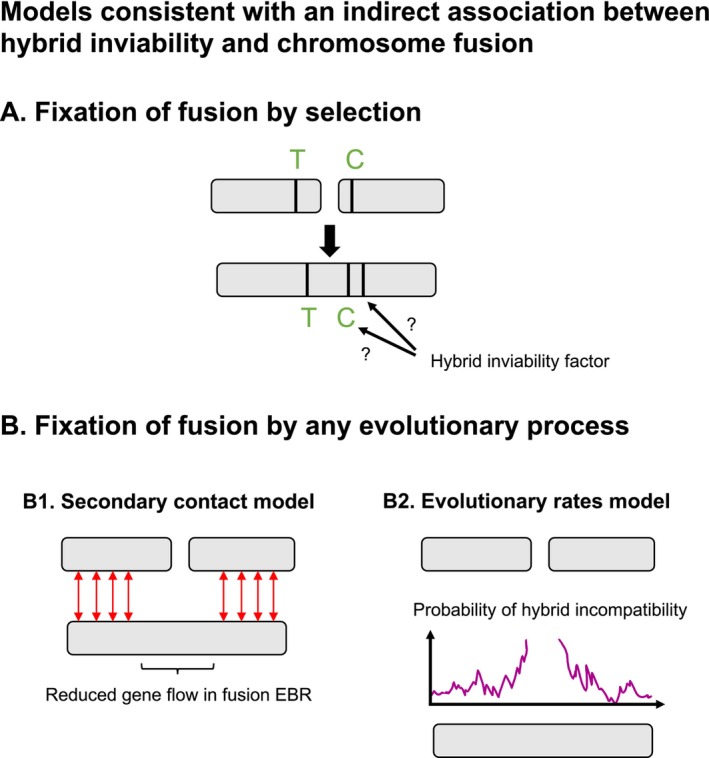
Models consistent with an indirect association between hybrid inviability and chromosome fusions. (A) Fusion of chromosomes containing loci involved in local adaptation (alleles T and C favoured in a certain environment). After the fusion, the two alleles have a higher probability of segregating together, which leads to fusions being indirectly favoured by selection (Guerrero and Kirkpatrick 2014). This model requires gene flow between the two populations, and that hybrid inviability evolves as a side effect of local adaptation, either because of pleiotropic effects of a locus affecting both local adaptation and inviability or because there is physical linkage between alleles at different loci affecting inviability and local adaptation, respectively. (B) In the following models, fusions get fixed in one population either by the process in (A) or driven by other evolutionary processes. (B1) Hybrid inviability evolves as a byproduct of substitutions and is enriched at fusions due to the low rate of purging at secondary contact (Noor et al. 2001; Rafajlović et al. 2021; Rieseberg 2001). Red arrows represent gene flow. (B2) A simple model that we propose here, where more divergent parts of the genome are more likely to contain hybrid incompatibilities—i.e., fixed variants or alleles with highly different allele frequencies in the two populations. A hypothetical probability curve for the presence of hybrid incompatibility loci along the chromosome(s) is given.

There are several models that could explain such an indirect relationship (physical linkage model) between chromosome fusions and hybrid inviability (Figure 5). An umbrella term for these models is the “recombination suppression” model, since they all require that fusions reduce recombination (Rieseberg 2001). First, a fusion can be favoured if loci involved in local adaptation are located on the chromosomes that fuse (Figure 5A). Hybrid inviability may then evolve as a side effect of the local adaptation. This model requires gene flow, which we also inferred in wood whites in the demographic history analysis (Figure 4). Thus, it is possible that recombination suppression between loci involved in local adaptation has contributed to the evolution of fusions and hybrid inviability in wood whites. However, mapping the genetic architecture of traits relevant to local adaptation would be needed to verify this observation. In addition, it would be important to investigate whether fusions were fixed by selection, drift, or a fixation bias (Mackintosh, Vila, Martin et al. 2023; Mackintosh, Vila, Laetsch et al. 2023). In female *L. sinapis* hybrids, meiotic drive favours the unfused state, lending support to the hypothesis that fusions in this system have fixed due to selection rather than drift (Boman et al. 2024). Second, if we condition on the fact that a fusion has fixed, then shifts in the recombination landscape can cause an enrichment of hybrid incompatibilities at fusions over evolutionary time (Figure 5B). In cases of gene flow, genomic regions that contain rearrangements that reduce the recombination rate in heterokaryotypes will maintain higher genetic differentiation, allowing for retention and evolution of genetic incompatibilities upon secondary contact (Noor et al. 2001; Rafajlović et al. 2021) (Figure 5B1). In another model that we propose here, faster lineage sorting in regions of low recombination will also increase the probability of enrichment of hybrid inviability factors mapping close to fusion breakpoints (Figure 5B2). In theory, hybrid incompatibilities could evolve in any region of the genome where novel mutations or sorting of ancestral variants differ between populations. However, incompatibilities are more likely to be observed in regions with higher substitution rates/faster lineage sorting, such as parts of chromosomes with low recombination rates. In agreement with this prediction, we observed that hybrid inviability candidate regions had higher *F*_ST_ and significantly lower recombination rates compared to non‐candidate regions. The difference in mean *F*_ST_ between candidate and non‐candidate regions was rather small (~0.013), but this is expected since the inferred candidate regions are much larger than the potential causative variants (which could be as small as a single nucleotide).

It is important to emphasise that one or more of the models may apply. The models are not mutually exclusive, and resolving their applicability and relative contributions to the *Leptidea* system and beyond will be challenging but will illuminate the role of chromosomal rearrangements in speciation. However, some clues on applicability and relative contributions can be garnered from the observed data. The reduced recombination observed for Fusion SWE chromosomes is consistent with all the recombination suppression models (Figure 5). Since the recombination was also reduced on the unfused (CAT) chromosomes of derived fusions, it is possible that the *Secondary contact* and *Evolutionary rates* models are important (Figure 5B). Even so, we did not find a significant enrichment of hybrid inviability candidate regions in low recombination regions of putatively older rearrangements (Unknown polarisation class; Table 1; Figure S9). This suggests that more recent rearrangements, such as the fusions in the SWE population, are more likely to be associated with hybrid inviability. This indicates that a low regional recombination rate by itself may not fully explain the association between fusions and hybrid inviability, lending support to the *Local adaptation* model (Figure 5A). To conclude, we find evidence for an indirect relationship between chromosomal rearrangements and hybrid inviability, probably driven by a reduced recombination in fused regions, but we cannot reject the possibility that several processes have contributed to the observed incompatibilities in the wood white butterflies.

### 4.3 Evolutionary History of *Leptidea sinapis*

The CAT and the SWE populations of *L. sinapis* represent the most extreme cases of intraspecific karyotype variation of any diploid animal. This striking variation in the karyotype setup is further characterised by a chromosome number cline, where populations in the southwestern part of the distribution range have the highest number of chromosomes, and populations in the northern (e.g., SWE) and eastern (e.g., Kazakhstan, KAZ) parts have the lowest. KAZ and SWE populations also have low genetic differentiation with an *F*_ST_ = 0.02 (Lukhtanov et al. 2011; Talla et al. 2019). Therefore, the SWE and CAT populations most likely represent an eastern and a western ancestry group, respectively. Lukhtanov et al. (2011) proposed that SWE and CAT *L*. sinapis diverged after the last glacial maximum (~20 kya). Our results suggest that the divergence is an order of magnitude older (> 400 kya). The remarkable chromosome number differences between *L. sinapis* populations have also been used to argue for a clinal speciation model (Lukhtanov et al. 2011; Templeton 1981). However, the relatively deep divergence time and inference of gene flow between the SWE and CAT populations indicate that the current chromosome number cline is a consequence of secondary contact. Hence, populations throughout central Europe and the British Isles likely have hybrid ancestry and constitute a transition zone where gene flow has occurred between ancestry groups. More detailed biogeographical analyses of *L. sinapis* in general and the central European populations in particular will be needed to verify the suggested hypothesis.

## Author Contributions

J.B., K.N., and N.B. designed the research. J.B., C.W. and N.B. performed research. J.B. analysed data and wrote the paper with input from K.N., R.V., C.W., and N.B.

## Conflicts of Interest

The authors declare no conflicts of interest.

## Benefit‐Sharing Statement

The benefit arising from this study is provided through the sharing of sequence data and analysis code, which are freely available and accessible to the public.

## Supporting information


Data S1.

## Data Availability

DNA‐sequencing data is available at the European Nucleotide Archive under study id PRJEB69278. Scripts are available in the Github repository: https://github.com/JesperBoman/Evolution‐of‐hybrid‐inviability‐associated‐with‐chromosome‐fusions.
