# Supplementary material for: Evolution of Hybrid Inviability Associated With Chromosome Fusions
Source: Mol Ecol. 2025 Feb 3;34(24):e17672. doi: 10.1111/mec.17672 (PMC12717967; doi:10.1111/mec.17672)
Supplement: Supplementary file 1 — Data S1. [file MEC-34-e17672-s001.pdf]

Supplementary Information

Figure S1: Syntenyplot between SWE, CAT and the Darwin Tree of Life (DToL) Asturian assemblies created using the *syntenyPlotteR* package in *R* based on LASTZ whole-genome alignments. Chromosomes are ordered by size except chromosome 48 in the DToL assembly. Chromosomes in the DToL assembly are colored by chromosome/rearrangement type. There are three Z sex chromosomes, which are not rearranged between these three genome assemblies.

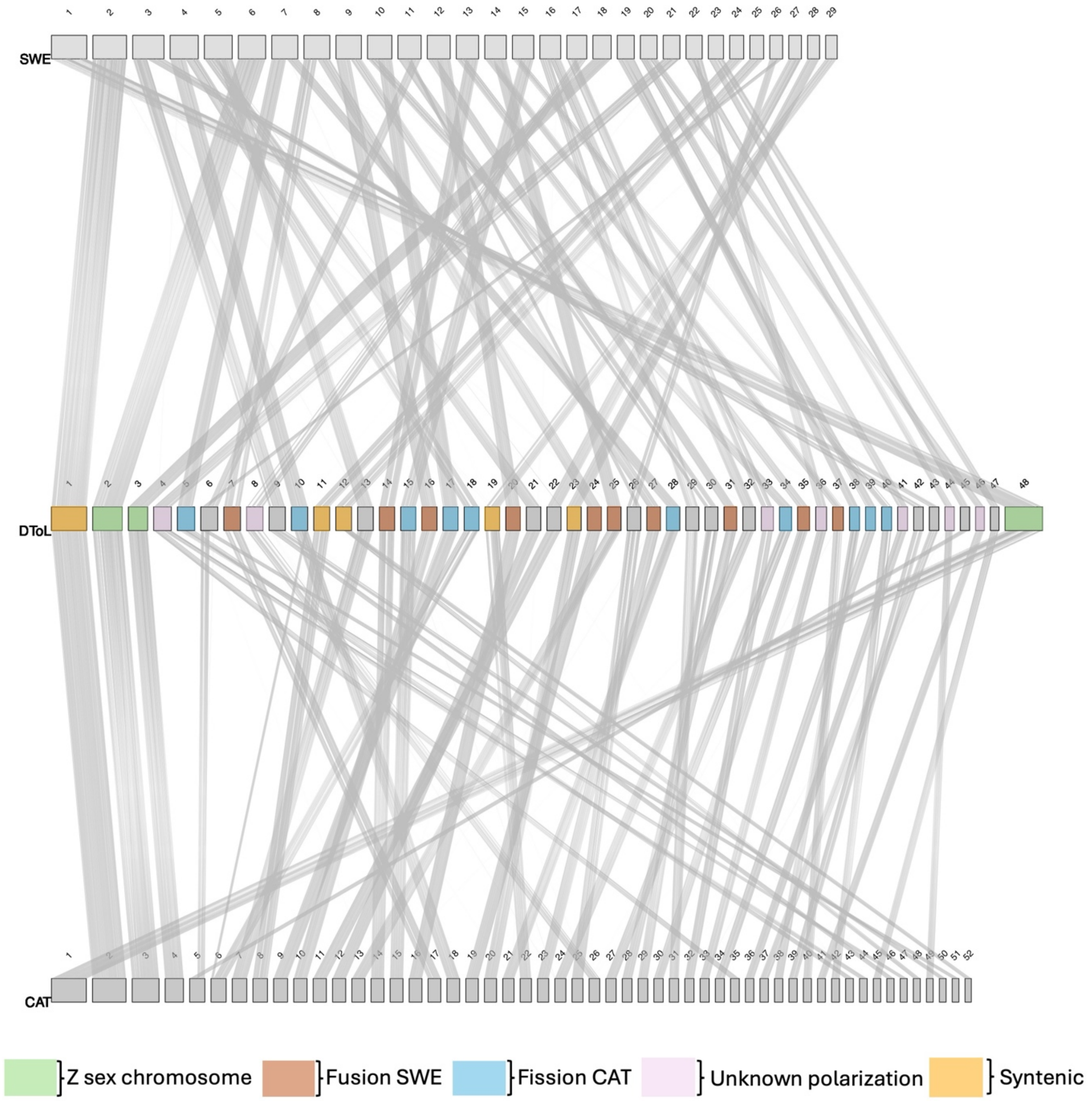

Figure S2: Illustration of how rearrangement polarity was determined by parsimony in Höök et al., (2023).

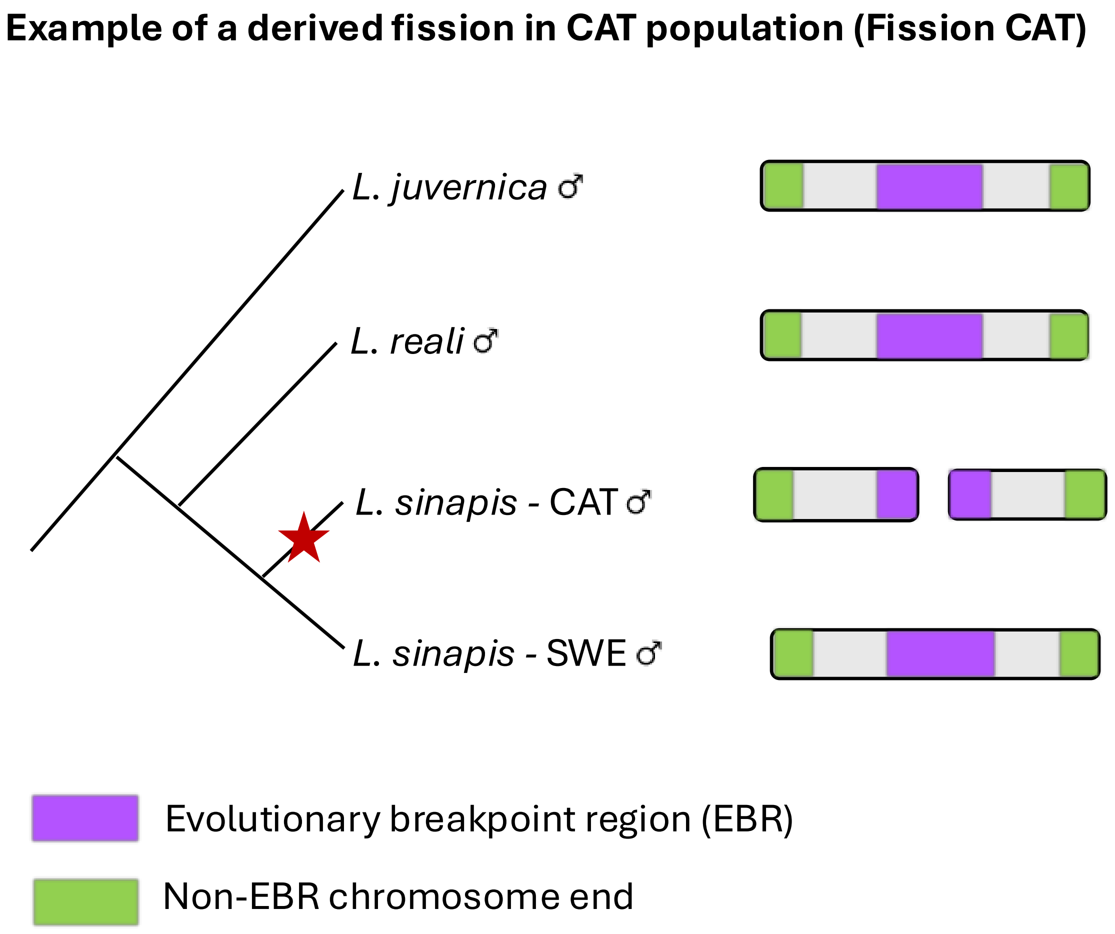

Figure S3: A pedigree tracing the ancestry of the F_2 population to the F_0 founders. Circles represent individuals and rectangles represent groups of individuals. For individuals, an identification code is provided, while digits for groups represent the number of females (orange), males (blue) and non-sexed (green) F_2 offspring for each cross.

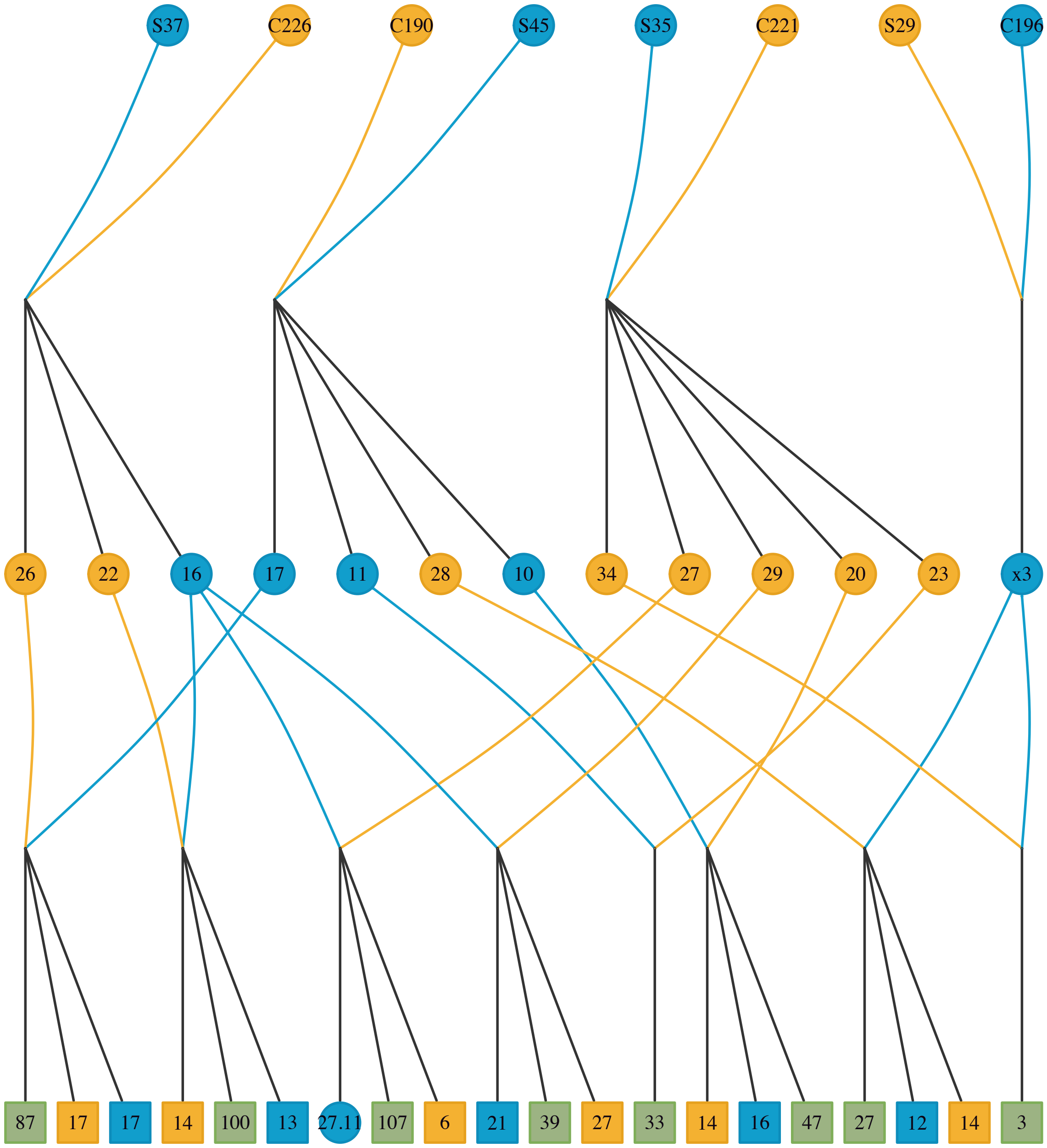

Figure S4: The narrow sense heritability (h^2) distributions for the additive survival model. (A) Using uninformative priors. (B) Using parameter-expanded priors. Both prior specifications showed similar results.

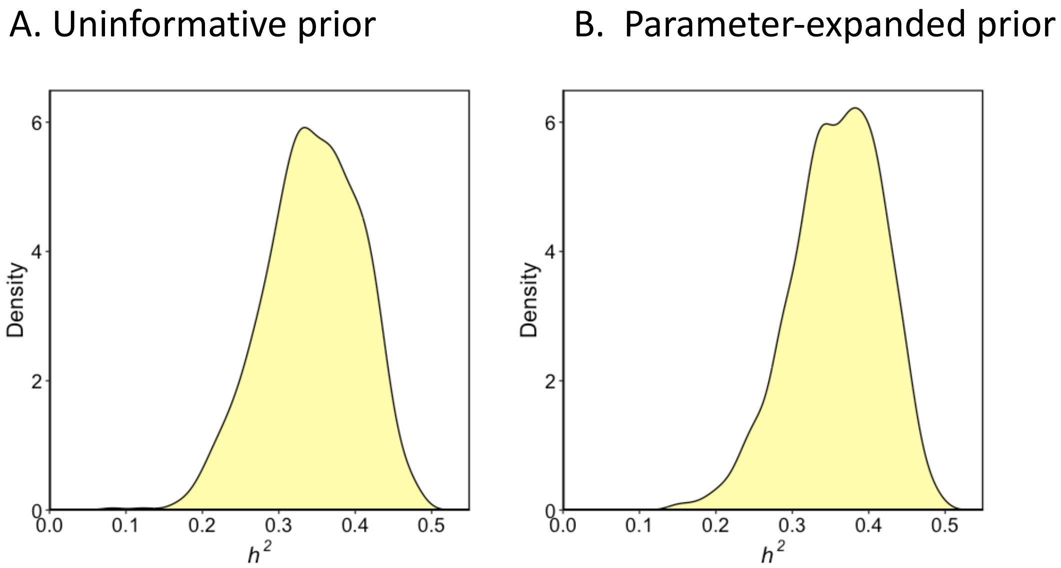

Figure S5. Allele frequency differences between the *Alive* and *Dead* pools as inferred by QTL-seq. Blue lines are the 90 %, 95 % and 99 % CI:s as determined by simulation. Yellow and red boxes represent regions were *Alive* has an excess of SWE and CAT alleles respectively. Excess was defined by having a smoothed allele frequency difference greater than the 95 % CI.

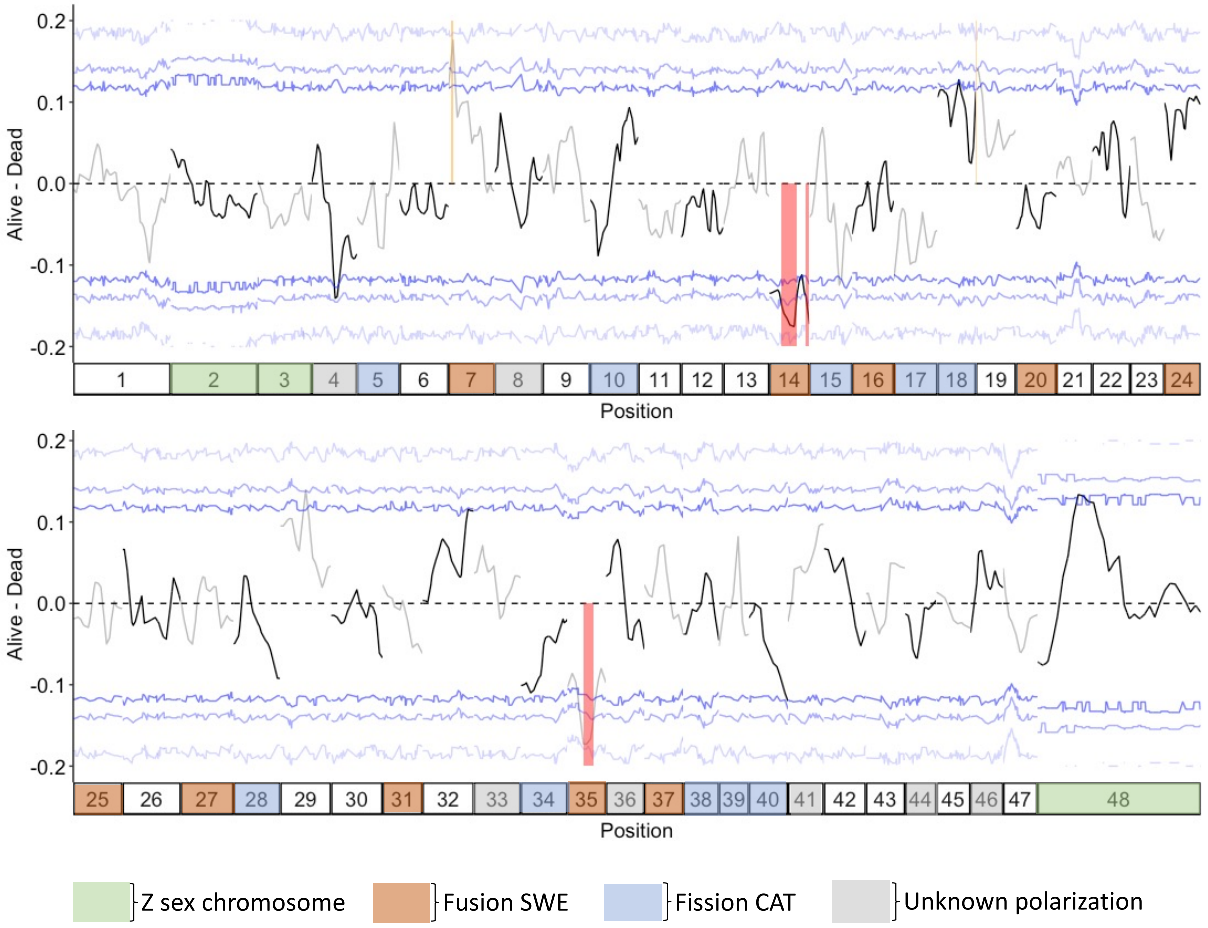

Figure S6. Allele frequency differences between the *Alive* pool and the egg pool. The resulting candidate regions significantly overlapped candidate regions obtained from the *Alive* vs *Dead* analysis. Loci have been polarized for the SWE allele frequency. Yellow and red boxes represent regions where the *Alive* pool has an excess of SWE and CAT alleles, respectively. The purple curve shows a generalized additive model that was fitted to the allele frequency differences between genomic regions. We defined candidate regions as stretches of chromosomes where the 95 % CI of the trajectory of the generalized additive model did not overlap an absolute allele frequency difference of 0.075. Chromosomes are plotted on a scale from first to last marker for each individual chromosome. Chromosomes 2, 3 and 48 are the Z-chromosomes. Chromosomes are ordered by size except for chromosome 48 which contains the ancestral Z-chromosome of Lepidoptera. Allele frequencies on Z-chromosomes were normalized by the sample sex ratio. The colors of chromosomes indicate if they represent derived fusions in the SWE population (brown), derived fissions in the CAT population (blue), or segregating fission/fusion polymorphisms (grey). Note that only simple rearrangements (involving two unfused elements) are shown.

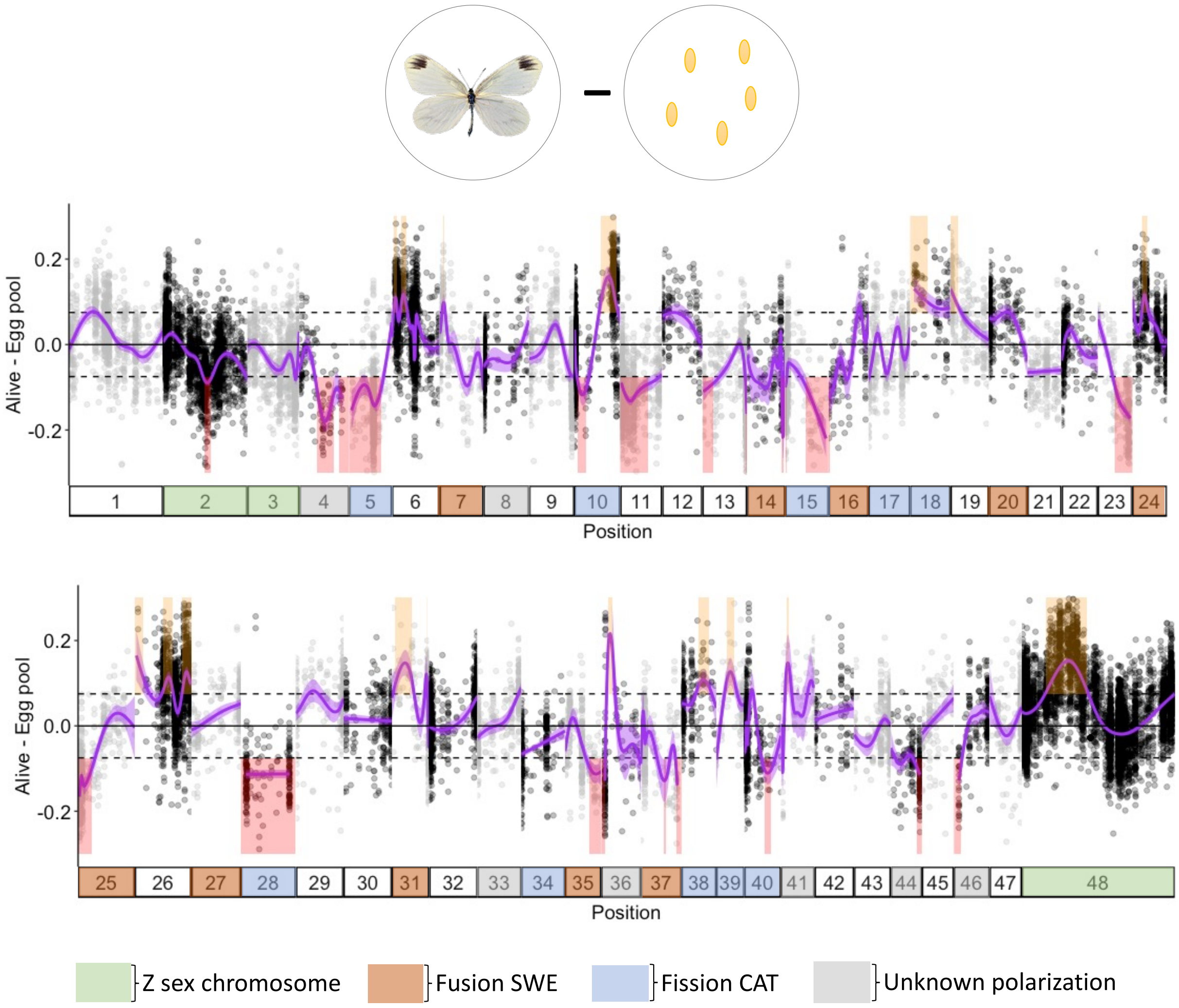

Figure S7: Investigation of the overlap between hybrid inviability candidate regions and EBRs and non-EBR chromosome ends for different window sizes: ±0.5, 1, 1.5, 2 and 3 Mb. For non-EBR ends the distance is positive (+). Across this range, the same result is obtained regardless of window size used. Significant *p*-values are still significant after Bonferroni correction per distance and type (EBR or non-EBR chromosome end).

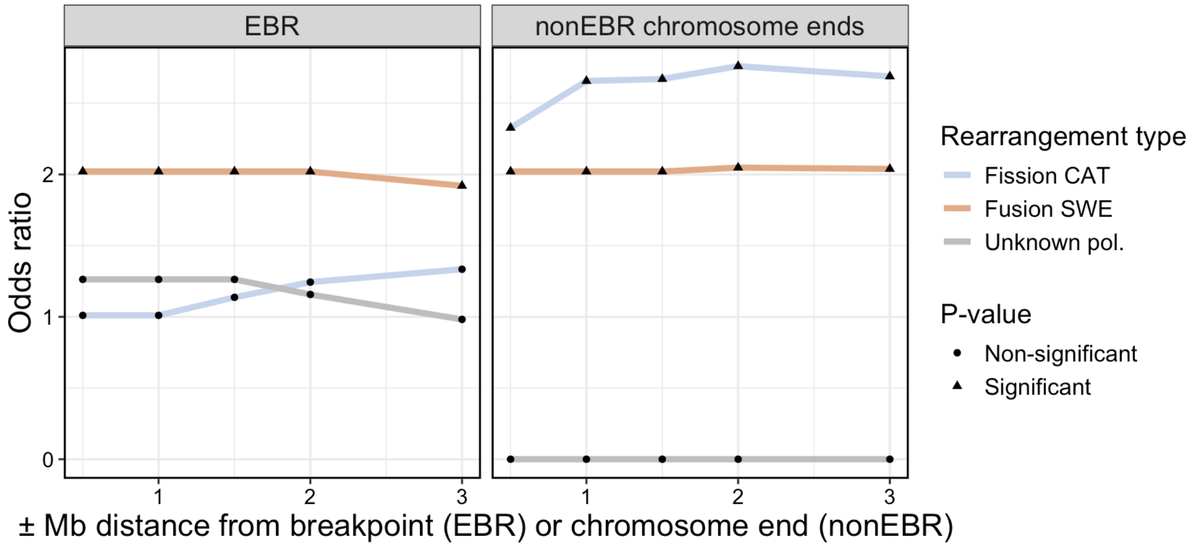

Figure S8: Parental population male recombination rates in candidate regions for hybrid inviability. (A) Recombination rates measured in centiMorgan per Mb (cM/Mb; extracted from pedigree-based linkage-maps) for all candidate regions. Lines connect the recombination rate values of each candidate region. Hair-cross symbols represent the genome-wide recombination rate in each population. Asterisks (*) indicate that recombination rates in the hybrid inviability candidate regions are significantly different from the genome-wide average as determined by resampling. (B) Distributions of the genome-wide recombination rates determined by resampling (100,000 replicates) for the SWE (orange) and the CAT (red) *L. sinapis* populations. The vertical solid and dashed lines show the observed average recombination rates of the candidate regions for the CAT and the SWE population, respectively.

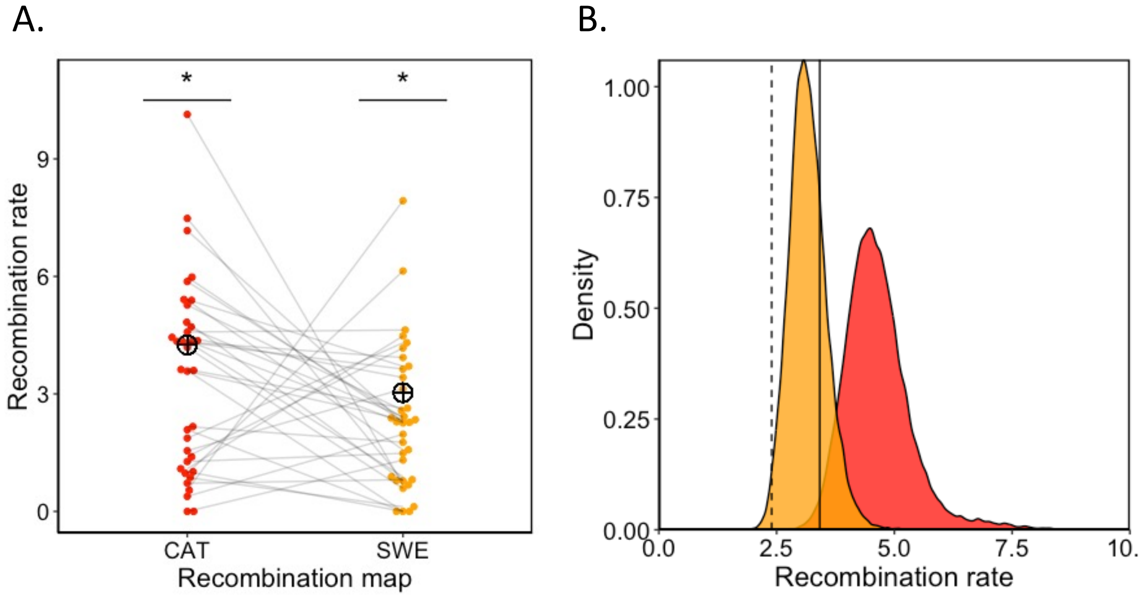

Figure S9: Patterns of recombination at rearranged chromosomes. Evolutionary breakpoint regions (EBRs) are shown in purple and non-EBR chromosome ends are shown in green. Patterns of average parental recombination rates in EBRs and non-EBRs chromosome ends are presented for ±1, 1.5, 2 and 3 Mb windows. For non-EBR ends the distance is positive (+). Error bars represent the standard error of the mean. Solid and dashed lines show the recombination rates in the CAT and the SWE population, respectively. Horizontal lines represent mean genome-wide recombination rates for the CAT (red) and SWE (orange) population.

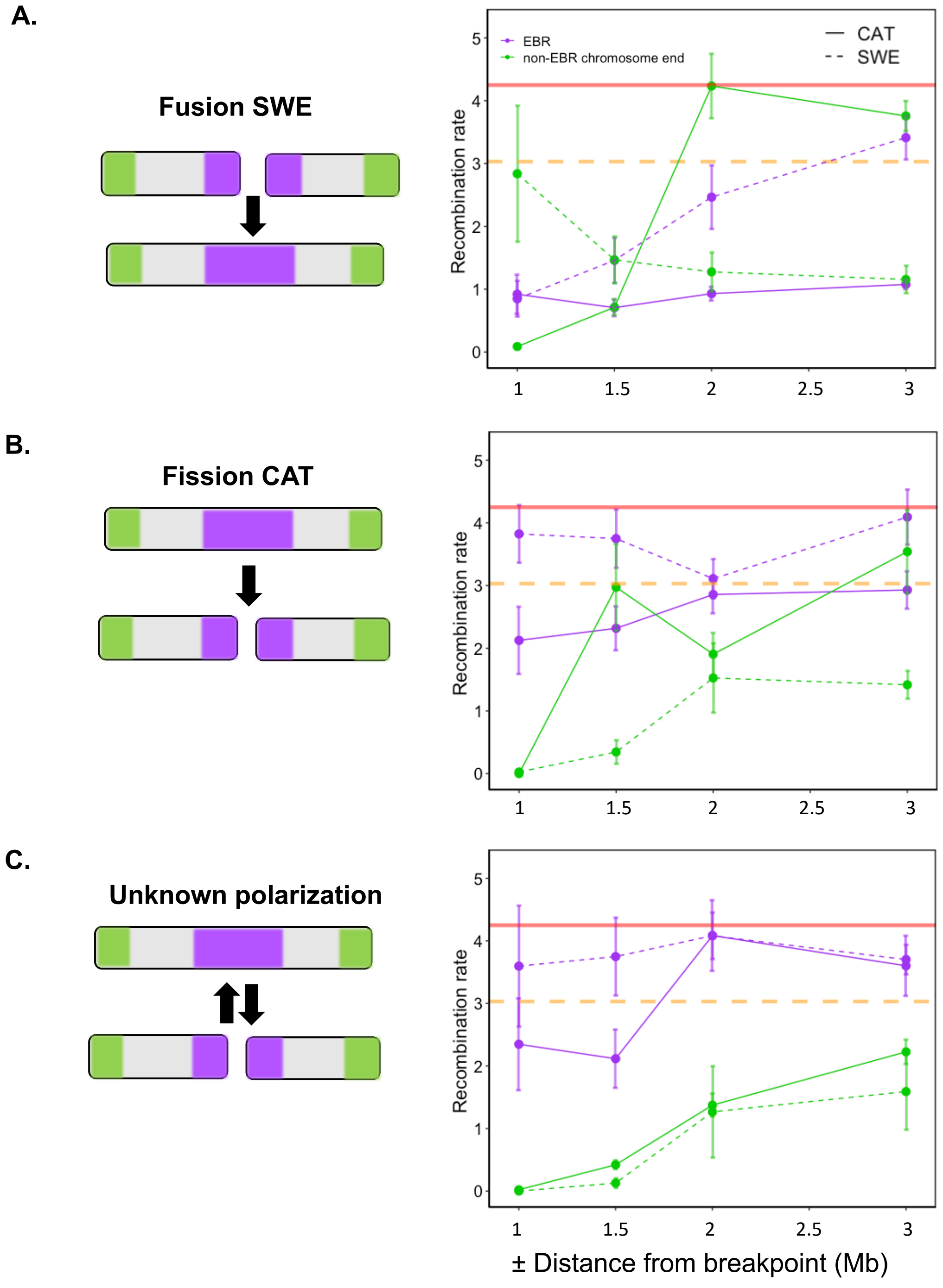

Figure S10: Model fit diagnostics for the Isolation-with-migration model for the demographic history of CAT and SWE *L. sinapis*. Top left is the observed joint minor-allele-frequency (MAF) spectrum. Top right is the joint MAF spectrum predicted by the model. Panels in the bottom row depict the residuals between “data” and “model”, per cell in the joint MAF (left) and as an overall histogram (right). The heatmap represents counts of SNPs in each cell of the joint MAF spectrum.

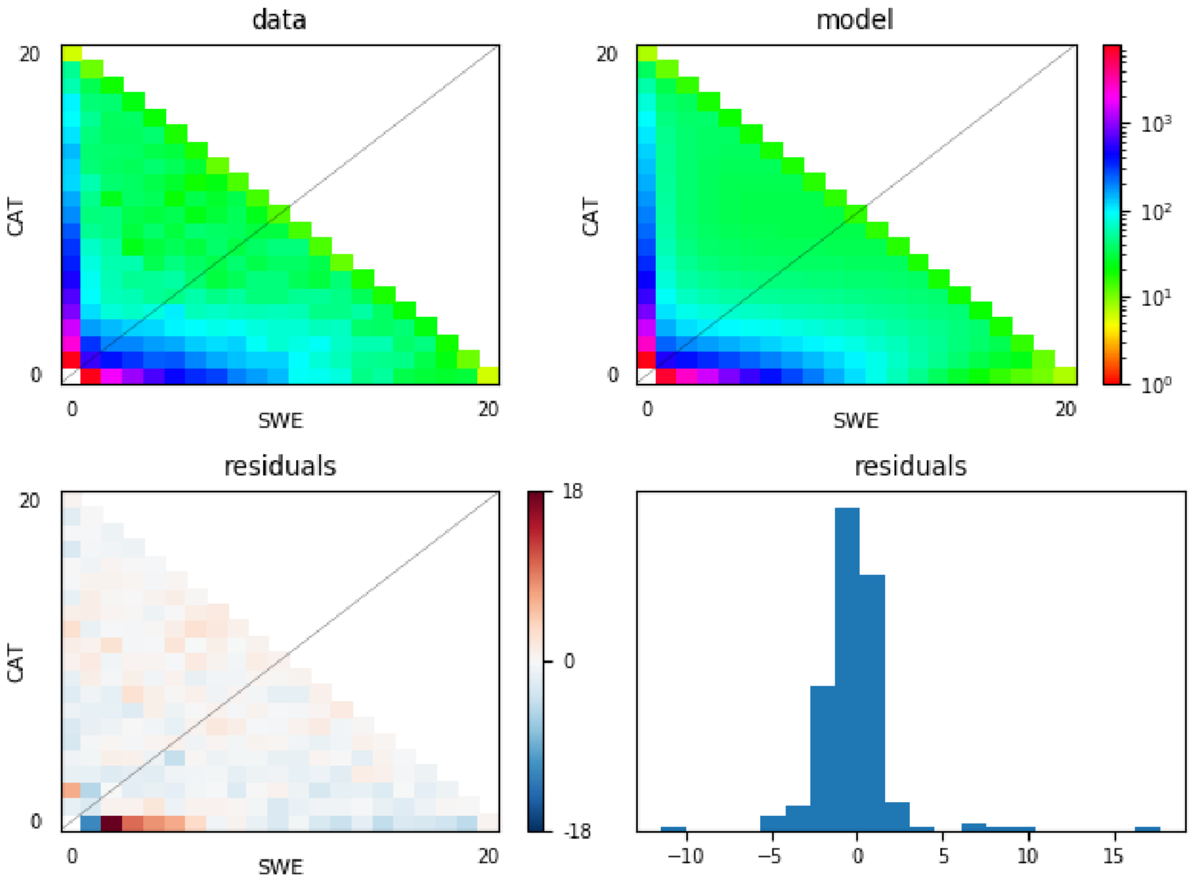

Figure S11: Population genetic summary statistics across all chromosomes measured in non-overlapping 10 kb windows. Values have been Z-transformed (subtracted by the mean and divided by the standard deviation) for ease of illustration. In addition, local regression curves (span=0.01, degree=1) were fit to the data, with shaded regions representing 95 % confidence intervals. Genetic differentiation (F_{ST}) is shown in blue, absolute divergence (D_{XY}) in dark green, average pairwise nucleotide diversity in the CAT population (π_{CAT}) in red and SWE (π_{SWE}) in orange respectively. Boxes represent hybrid inviability candidate regions (see Figure 2 of main text). Of note, large peaks of D_{XY} and π on chromosomes 11, 12 and 42 possibly represent collapsed assembly regions due to an increased coverage among some *L. sinapis, L. juvernica* and *L. reali* (data not shown).

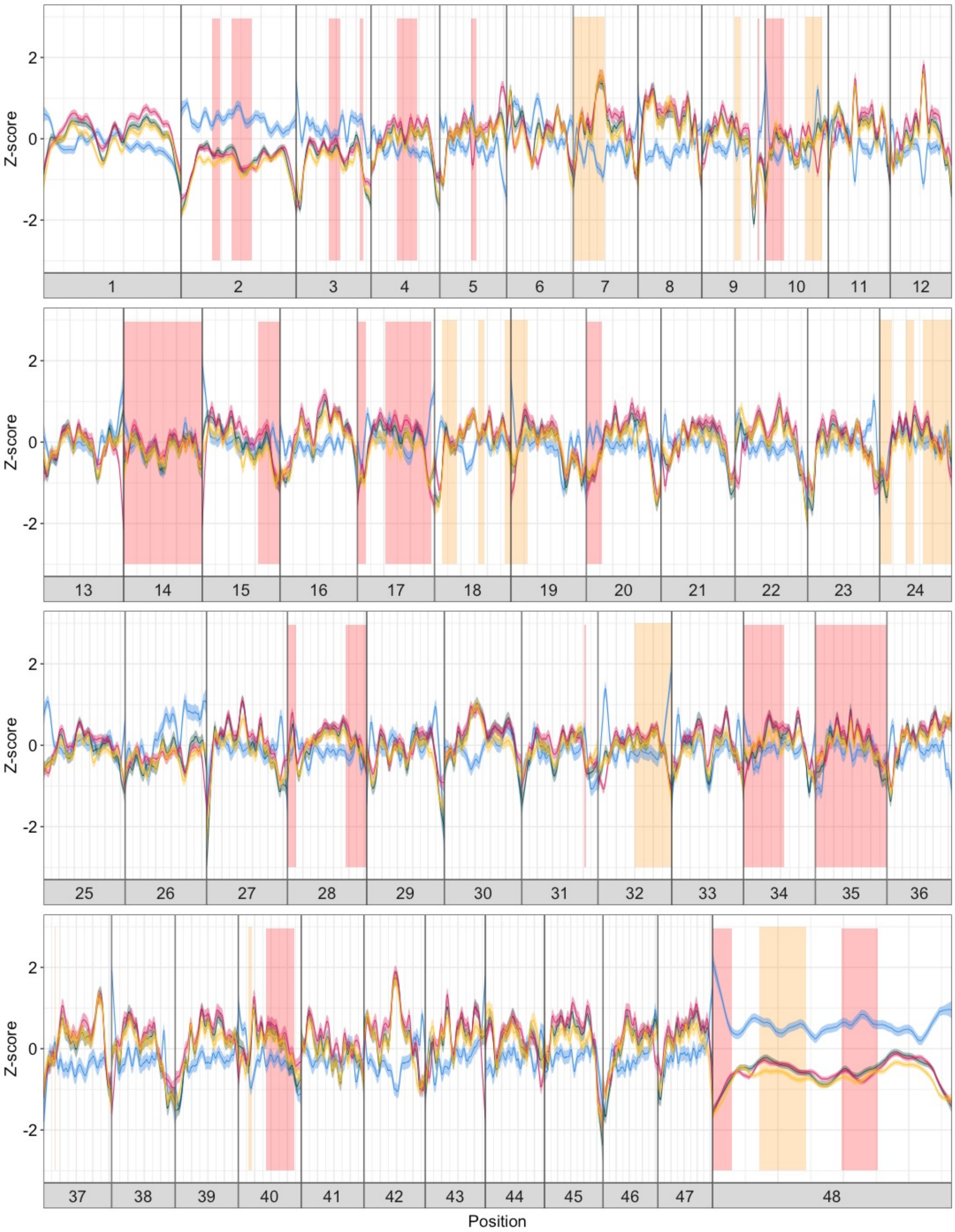

Table S1. Information about sequencing pools. (A) Summary of sequencing statistics for the pool-seq libraries and sex ratio of pools. Sex ratios (expressed as % of males) of egg pool, dead embryos and dead larvae and pupae were inferred based on read mapping coverage of chromosome 48 (Z_1). F_2 adults were sexed phenotypically. (B) Extraction pools and DNA concentrations.

A. Sequencing data

Pool	Sample size	% Males	Pool type	Million reads	Percentage phred score >=Q30(%)	Average read depth with MQ>20*	Variance of read depths with MQ>20*
Egg pool	73	32.2%	Mix	393.26	91.38	115x	7654
Dead embryos	298	50.5%	Dead	391.33	91.5	119x	8119
Dead larvae and pupae	72	37.5%	Dead	383.13	91.33	115x	8221
F_2 adult females	80	0%	Alive	398.72	91.24	115x	10943
F_2 adult males	77	100%	Alive	418.98	91.61	137x	7848

*At fixed difference marker loci. MQ = mapping quality

B. Extraction data

Sample	Num. inds. co-extracted	Qubit (ng/uL)	Sequencing pool
20RP1	5	35.2	Egg pool
20RP2	5	43.1	Egg pool
22RP1	5	20.5	Egg pool
22RP2	5	43.1	Egg pool
23RP1	5	1.81	Egg pool
23RP2	5	1.98	Egg pool
26RP1	5	41.9	Egg pool
26RP2	5	5.11	Egg pool
27RP1	5	5.02	Egg pool
27RP2	5	11.6	Egg pool
28RP1	5	38	Egg pool
28RP2	5	21.6	Egg pool
29RP1	5	42.7	Egg pool
29RP2	5	28.4	Egg pool
34RP	3	2.59	Egg pool
20.13	1	5.14	F_2 adult females
20.14	1	20.9	F_2 adult females
20.15	1	8.69	F_2 adult females
20.16	1	1.98	F_2 adult females
20.18	1	2.15	F_2 adult females
20.22	1	15.6	F_2 adult females
20.3	1	26.8	F_2 adult females
20.33	1	11.9	F_2 adult females
20.6	1	6.09	F_2 adult females
20.9	1	7.18	F_2 adult females
22.16	1	1.31	F_2 adult females
22.18	1	17.2	F_2 adult females
22.21(2)	1	49.4	F_2 adult females
22.23	1	1.25	F_2 adult females

22.3	1	29.5	F_2 adult females
22.37	1	20.7	F_2 adult females
22.4	1	7.53	F_2 adult females
22.42	1	9.5	F_2 adult females
22.46	1	10.4	F_2 adult females
22.5	1	5.92	F_2 adult females
22.50	1	25.4	F_2 adult females
22.7	1	10.9	F_2 adult females
22.8	1	1.17	F_2 adult females
26.1	1	19.6	F_2 adult females
26.10	1	48.6	F_2 adult females
26.15	1	46.9	F_2 adult females
26.19	1	36.6	F_2 adult females
26.21	1	3.77	F_2 adult females
26.25	1	2.62	F_2 adult females
26.28	1	50.2	F_2 adult females
26.3(2)	1	27.3	F_2 adult females
26.31	1	8.3	F_2 adult females
26.35	1	61.9	F_2 adult females
26.46	1	24.7	F_2 adult females
26.47	1	4.37	F_2 adult females
26.5(3)	1	6.39	F_2 adult females
27.1	1	4.82	F_2 adult females
27.13	1	5.14	F_2 adult females
27.2	1	7.4	F_2 adult females
27.6	1	21.3	F_2 adult females
27.8	1	44	F_2 adult females
28.1	1	11.1	F_2 adult females
28.10(2)	1	64.8	F_2 adult females
28.12	1	18.3	F_2 adult females
28.15	1	22.7	F_2 adult females
28.19	1	30.8	F_2 adult females
28.20	1	3.78	F_2 adult females
28.21	1	46.4	F_2 adult females
28.22	1	48.4	F_2 adult females
28.23	1	26.8	F_2 adult females
28.24	1	42	F_2 adult females
28.25	1	8.9	F_2 adult females
28.26	1	20.4	F_2 adult females
28.27	1	23.1	F_2 adult females
28.7	1	34.3	F_2 adult females
29.(38?)	1	2.96	F_2 adult females
29.(40?)	1	24.8	F_2 adult females
29.(42?)	1	54.9	F_2 adult females
29.12	1	16.7	F_2 adult females
29.14	1	13.9	F_2 adult females
29.15	1	14.1	F_2 adult females
29.17	1	19.4	F_2 adult females
29.30	1	10.6	F_2 adult females
29.31	1	67.3	F_2 adult females
29.32	1	19.6	F_2 adult females
29.33	1	15.5	F_2 adult females
29.33	1	75	F_2 adult females
29.35	1	49.4	F_2 adult females
29.41	1	22.7	F_2 adult females
29.44	1	17.1	F_2 adult females
29.45	1	3.68	F_2 adult females
29.47	1	20.8	F_2 adult females

29.48	1	25.8	F_2 adult females
29.49	1	76	F_2 adult females
29.51	1	20	F_2 adult females
29.52	1	6.28	F_2 adult females
29.57	1	37	F_2 adult females
29.58	1	27.9	F_2 adult females
29.61	1	30.5	F_2 adult females
29.7	1	8.39	F_2 adult females
20.10	1	62.4	F_2 adult males
20.17	1	6.13	F_2 adult males
20.2	1	47	F_2 adult males
20.26	1	25.9	F_2 adult males
20.27(2)	1	40.3	F_2 adult males
20.30	1	27.5	F_2 adult males
20.31	1	17	F_2 adult males
20.32	1	41.2	F_2 adult males
20.35	1	6.05	F_2 adult males
20.36	1	1.87	F_2 adult males
20.37	1	13.7	F_2 adult males
20.4	1	3.67	F_2 adult males
20.8	1	30.8	F_2 adult males
22.10	1	12.6	F_2 adult males
22.12	1	15.2	F_2 adult males
22.13	1	2.96	F_2 adult males
22.14	1	33.3	F_2 adult males
22.22	1	8.25	F_2 adult males
22.30	1	21.9	F_2 adult males
22.35(2)	1	32.3	F_2 adult males
22.40	1	13.7	F_2 adult males
22.43	1	17.6	F_2 adult males
22.49	1	9.53	F_2 adult males
22.9	1	9.33	F_2 adult males
26.14	1	62.3	F_2 adult males
26.16	1	7.31	F_2 adult males
26.18	1	32.2	F_2 adult males
26.19	1	1.56	F_2 adult males
26.2	1	13.8	F_2 adult males
26.24	1	7.86	F_2 adult males
26.30	1	27.9	F_2 adult males
26.32	1	3.66	F_2 adult males
26.33	1	67.6	F_2 adult males
26.34	1	20.6	F_2 adult males
26.36	1	24.3	F_2 adult males
26.37	1	6.12	F_2 adult males
26.45	1	26.8	F_2 adult males
26.49	1	56.3	F_2 adult males
26.6	1	22.4	F_2 adult males
26.7	1	72.6	F_2 adult males
26.8	1	19.4	F_2 adult males
27.11	1	30.3	F_2 adult males
27.14	1	1.45	F_2 adult males
28.11	1	2.6	F_2 adult males
28.14	1	6.27	F_2 adult males
28.17	1	36.2	F_2 adult males
28.18(2)	1	31.6	F_2 adult males
28.28	1	11.2	F_2 adult males
28.3	1	76.8	F_2 adult males
28.4	1	8.6	F_2 adult males

28.5	1	7.05	F_2 adult males
28.6	1	50.3	F_2 adult males
28.9	1	12.1	F_2 adult males
29.(39?)	1	43.5	F_2 adult males
29.11	1	21	F_2 adult males
29.13	1	57.2	F_2 adult males
29.19	1	7.1	F_2 adult males
29.21	1	12.2	F_2 adult males
29.22	1	40.1	F_2 adult males
29.24	1	3.33	F_2 adult males
29.25(2)	1	73.6	F_2 adult males
29.26	1	31	F_2 adult males
29.27	1	29.1	F_2 adult males
29.28	1	12.9	F_2 adult males
29.29	1	5.41	F_2 adult males
29.3	1	3.91	F_2 adult males
29.36	1	8.45	F_2 adult males
29.37	1	15.8	F_2 adult males
29.49	1	9.08	F_2 adult males
29.5	1	7.29	F_2 adult males
29.53	1	18.8	F_2 adult males
29.54	1	29.2	F_2 adult males
29.59	1	29.6	F_2 adult males
29.6	1	7.79	F_2 adult males
29.62	1	17.8	F_2 adult males
29.8	1	8.36	F_2 adult males
20.29DP2	1	6.63	Dead larvae and pupae
22.1DPI2	1	4.04	Dead larvae and pupae
22.44DP2	1	8.34	Dead larvae and pupae
26.12DP2	1	18.5	Dead larvae and pupae
26.30DVP2	1	17.6	Dead larvae and pupae
29.4DL2	1	10.8	Dead larvae and pupae
29.10DL2	1	7.22	Dead larvae and pupae
20.21DL	1	23.45	Dead larvae and pupae
22.27DL	1	34.2	Dead larvae and pupae
26.17DL	1	29.4	Dead larvae and pupae
26.26DL	1	10.35	Dead larvae and pupae
28.8DL	1	36.95	Dead larvae and pupae
20(1)DL	6	12.9	Dead larvae and pupae
27(1)DL	6	14.6	Dead larvae and pupae
28(1)DL	2	2.5	Dead larvae and pupae
22(1)DL	5	11.1	Dead larvae and pupae
22(2)DL	6	10.2	Dead larvae and pupae
22(3)DL	5	11.8	Dead larvae and pupae
22(4)DL	5	7.88	Dead larvae and pupae
26(1)DL	6	11.1	Dead larvae and pupae
26(2)DL	6	13.6	Dead larvae and pupae
26.9DL	1	78.05	Dead larvae and pupae
29(1)DL	9	19.8	Dead larvae and pupae
29.16DL	1	6.405	Dead larvae and pupae
20.20D.P/I	1	23.3	Dead larvae and pupae
26.4DP2	1	9.43	Dead larvae and pupae
20(1)D.E	16	17.5	Dead embryos
20(2)D.E	11	19.4	Dead embryos
22(1)D.E	11	25	Dead embryos
22(2)D.E	11	5.96	Dead embryos
22(3)D.E	14	17.7	Dead embryos
22(4)D.E	11	8.88	Dead embryos

22(5)D.E	13	16.2	Dead embryos
22(6)D.E	6	11.3	Dead embryos
23(1)D.E	10	1	Dead embryos
23(2)D.E	13	1	Dead embryos
26(2)D.E	10	2.23	Dead embryos
26(3)D.E	14	14.3	Dead embryos
26(4)D.E	9	4.04	Dead embryos
26(5)D.E	12	12.1	Dead embryos
26(6)D.E	17	4.05	Dead embryos
27(1)D.E	5	5.24	Dead embryos
27(2)D.E	10	37	Dead embryos
27(3)D.E	13	16.7	Dead embryos
27(4)D.E	5	23.1	Dead embryos
27(5)D.E	15	7.17	Dead embryos
27(6)D.E	21	39.5	Dead embryos
27(7)D.E	5	5.87	Dead embryos
27(8)D.E	17	5.61	Dead embryos
28(1)D.E	9	6.54	Dead embryos
28(2)D.E	5	5.79	Dead embryos
29(1)D.E	6	13.4	Dead embryos
29(2)D.E	9	90.2	Dead embryos

Table S2. Filtering parameters for population-resequencing data to obtain a set of high-quality SNPs.

**Parameter**	**Filtering threshold**
Fisher strand bias	<60
Strand odds ratio	<3
Mapping quality	>40
Mapping quality rank sum test	>-12.5
Quality by depth	>2
Read position rank sum test	>-8
Depth	<31.09 (3 standard deviations above mean read coverage)
Max missing	1 (no missing allele information)

Table S3. Survival model. Uninformative prior (*V* = 1, *nu* = 1^{-6}). 100,000 iterations. 10,000 iterations discarded as burn-in. Thinning interval = 100, yielding 900 samples.

**Variable**	**Posterior mean [95 % credible interval]**	**Effective sample size**	**pMCMC**
Intercept	-0.7568 [-2.2180 – 0.8066]	900	0.322
Animal	3.08 [1.073 – 5.166]	526.7	N/A

Table S4. Survival model. Parameter-expanded prior (*V* = 1, *nu* = 1, *alpha.mu* = 0, *alpha.V* =1000). 100,000 iterations. 10,000 iterations discarded as burn-in. Thinning interval = 100, yielding 900 samples.

**Variable**	**Posterior mean [95 % credible interval]**	**Effective sample size**	**pMCMC**
Intercept	-0.7836 [-2.4437 – 0.7604]	900	0.331
Animal	3.384 [1.497 – 5.756]	761.5	N/A

Table S5. Development time model with parameter-expanded priors*. Using Z-scores of development times. 100,000 iterations. 10,000 iterations discarded as burn-in. Thinning interval 100, yielding 900 samples. Family Gaussian.

**Variable**	**Posterior mean [95 % credible interval]**	**Effective sample size**	**pMCMC**
Intercept	-1.72100 [-2.19377 – -1.20066]	343.9	< 0.001
SexMale	-0.02072 [-0.08679 – 0.03877]	900.0	0.13111
SurvivalAlive	-0.27237 [-0.44827 – -0.11510]	587.0	< 0.001
Animal	0.01061 [8.873e-08– 0.04328]	97.52	N/A
Units	0.00114 [0.01459– 0.01771]	900	N/A

* Parameter-expanded prior command*: list(R = list(V = 1, nu = 0.002), G = list(G1 = list(V = diag(6), nu = 0.002, alpha.mu = rep(0, 6), alpha.V= diag(1, 6, 6)), G2 = list(V = 1, nu = 0.002, alpha.mu = 0, alpha.V = 1)))*

Table S6. Development time model with uninformative prior*. Using Z-scores of development times. 100,000 iterations. 10,000 iterations discarded as burn-in. Thinning interval 100, yielding 900 samples. Family Gaussian.

**Variable**	**Posterior mean [95 % credible interval]**	**Effective sample size**	**pMCMC**
Intercept	-1.31226 [-1.57881– -0.99039]	16.71	< 0.001
SexMale	-0.02283 [-0.08696 – 0.04056]	900	0.467
SurvivalAlive	-0.31697 [-0.46309 – -0.18015]	900	< 0.001
Animal	0.04992 [0.0001489- 0.07947]	13.32	N/A
Units	0.01616 [0.01466 – 0.01791]	241.6	N/A

* Uninformative prior command*: list(R = list(V = 1, nu = 1e-6), G = list(G1 = list(V = diag(6), nu = 1e-6), G2 = list(V = 1, nu = 1e-6)))*

Table S7. Comparison between different methods to detect allele frequency shifts between *Alive* and *Dead* group of pools. Note that these methods are not intended to be completely equal. The Kolmogorov-Smirnov (KS) test was here used as a test per an entire chromosome while the GAM analysis and QTLseqr highlights specific candidate regions per chromosome. (A) the pairwise Pearson correlation coefficient between different methods. Chromosomes with a significant test for a certain method was coded as "1" and non-significant chromosomes were coded as "0". The *p*-value was $9*10^{-5}$ between KS and GAM, while none of the other methods were significantly correlated with the more conservative QTL-seq. (B) Chromosomes with significant (green) and nonsignificant (red) allele frequencies differences between *Alive* and *Dead* per method. See Figure 2 of the main manuscript and Figure S1 for more information about chromosome categories.

A.

	**KS**	**GAM**	**QTLSeqr**
**KS**	1	0.53 ***	0.24 N.S.
**GAM**		1	0.25 N.S.
**QtlSeqr**			1

B.

**Chromosome**	**Number of markers**	**KS**	**GAM**	**QTLSeqr**
1	876	green	red	red
2	2319	green	green	red
3	945	green	green	red
4	137	red	green	red
5	486	green	green	red
6	1265	green	red	red
7	259	green	green	green
8	354	red	red	red
9	792	green	green	red
10	1272	green	green	red
11	925	green	red	red
12	174	green	red	red
13	1076	red	red	red
14	166	green	green	green
15	631	green	green	red
16	129	red	red	red
17	461	green	green	red
18	108	green	green	red
19	291	green	green	green
20	324	green	green	red
21	211	red	red	red
22	305	green	red	red
23	210	green	red	red

24	581			
25	411			
26	1209			
27	213			
28	191			
29	319			
30	286			
31	283			
32	1079			
33	297			
34	294			
35	261			
36	140			
37	264			
38	312			
39	91			
40	505			
41	78			
42	110			
43	379			
44	202			
45	125			
46	204			
47	264			
48	5906			

Table S8. Associations between chromosomal rearrangements and candidate regions for hybrid inviability when using the QTL-seq method to identify candidate regions. The analysis was performed for the entire chromosomes, evolutionary breakpoint regions (EBRs) and non-EBR ends of chromosomes, respectively. For full chromosome and chromosome ends we also investigated syntenic chromosomes.

**Category**	**Polarization**	**Odds ratio**	***p*-value**	***p*-value***
Chromosome	Fission CAT	0	0.229	0.916
Chromosome	Fusion SWE	4.871	0.002	0.008
Chromosome	Unknown	0	0.407	1
Chromosome	Syntenic	0.332	0.450	1
EBR	Fission CAT	0	0.854	1
EBR	Fusion SWE	0	0.875	1
EBR	Unknown	0	0.899	1
non-EBR ends	Fission CAT	0	0.884	1
non-EBR ends	Fusion SWE	0	0.878	1
non-EBR ends	Unknown	0	0.901	1
Chromosome ends	Syntenic	1.547	0.238	0.952

*Corrected for multiple testing using the Bonferroni method for each category separately.

Table S9. Analysis of variance (ANOVA) of coverage between chromosome types and pools. See Figure 3A in the main manuscript for the pools and chromosome types considered. The model used was *Coverage ~ Chromosome type * Pool* (i.e. including an interaction effect). A significant difference between chromosome types was observed when including Z chromosomes but that significance disappeared when running the model on a dataset where Z chromosomes were excluded.

**Response variable**	**Predictor variable**	***F***	***p***	**Model**
Coverage	Chromosome type	2.480	0.033	Including Z's
	Pool	1.881	0.115	
	Chromosome type x Pool	1.283	0.193	
Coverage	Chromosome type	0.477	0.752	Excluding Z's
	Pool	2.275	0.063	
	Chromosome type x Pool	0.079	1	

F and *p* were rounded to three decimals.

Table S10. Wilcoxon tests of recombination rate differences at fusion EBRs (±1 Mb of breakpoint) compared to genome-wide rates (one-sample Wilcoxon test) and non-EBR chromosome ends of chromosomes involved in rearrangements (two-sample Wilcoxon test). We tested both SWE and CAT parental population recombination rates. EBRs show significantly lower recombination rates than genome-wide but are not significantly different from non-EBRs, regardless of recombination map. Significant values are highlighted in bold.

Test	Polarization	Recombination map	*p*
EBR vs genome-wide	Fusion SWE	SWE	**0.02009**
EBR vs genome-wide	Fusion SWE	CAT	**0.02833**
EBR vs non-EBR	Fusion SWE	SWE	0.2612
EBR vs non-EBR	Fusion SWE	CAT	0.6699

Table S11 Wilcoxon tests of recombination rate differences at EBRs (±1 Mb of breakpoint) of fissions and rearranged chromosomes with unknown polarization, compared to genome-wide rates (one-sample Wilcoxon tests) and non-EBR chromosome ends of chromosomes involved in rearrangements (two-sample Wilcoxon tests), using a SWE and CAT parental population recombination map respectively. Recombination rates at EBRs for these chromosomes are roughly equal to genome-wide rates but generally higher than their more recombinationally quiescent non-EBR ends. Significant values are highlighted in bold.

**Test**	**Polarization**	**Recombination map**	***p***
EBR vs genome-wide	Fission CAT	SWE	0.4922
EBR vs genome-wide	Unknown	SWE	1
EBR vs genome-wide	Fission CAT	CAT	0.2685
EBR vs genome-wide	Unknown	CAT	0.2785
EBR vs non-EBR	Fission CAT	SWE	**0.001163**
EBR vs non-EBR	Unknown	SWE	**0.005494**
EBR vs non-EBR	Fission CAT	CAT	0.06639
EBR vs non-EBR	Unknown	CAT	0.1956

Table S12: Inference of the demographic history of SWE and CAT *L. sinapis.* Two models were fit: an isolation model assuming no migration since divergence and isolation-with-migration. The latter model provided a superior fit as evaluated by AIC. Numbers in brackets show the 95 % confidence interval for relevant parameters.

	**Isolation**	**Isolation-with-migration**
**AIC**	6218	2480
**Number of parameters**	4	6
**Log likelihood**	-3105.29	-1245.94
T	127,652 [-70,918 – 212,753]	403,329 [112,035 – 672,215]
**Proportion of split**	0.86 [0.8 –1.0]	0.13 [-0.1 – 0.4]
N_{ANC}	354,588	280,418
N_{CAT}	450,792 [35,589 – 957,387]	578,270 [196,500 – 954,426]
N_{SWE}	1,924,429 [1,560,187 – 2,269,363]	396,180 [112,391 – 674,349]
$M_{SWE \rightarrow CAT}$	NA	1.07 [0.5 –1.6]
$M_{CAT \rightarrow SWE}$	NA	0.18 [0 – 0.4]

Table S13. Statistical analysis of population genetic summary statistics. Results from the ANOVA analysis of differences between population genetic summary statistics (estimated in 10 kb windows) inside and outside hybrid inviability candidate regions. Chromosome and Status (within or outside candidate regions) represent the fixed effect predictors.

Response variable	Predictor variable	*F*	*p*
F_{ST}	Chromosome	131.43	$< 2.2*10^{-16}$
	Status	43.68	$3.895 *10^{-11}$
D_{XY}	Chromosome	105.005	$< 2.2*10^{-16}$
	Status	1.290	0.256
π_{SWE}	Chromosome	108.969	$< 2.2*10^{-16}$
	Status	0.0694	0.79
π_{CAT}	Chromosome	76.794	$< 2.2*10^{-16}$
	Status	23.724	$1.114 *10^{-6}$

F and *p* were rounded to three decimals.

Text S1: Measuring the false positive rate of the *Alive* vs *Dead* GAM analysis

Background

To detect the genetic basis of hybrid inviability we first used the Kolmogorov-Smirnov (KS) test per chromosome to detect differences in allele frequency distributions between the Alive and Dead group of pools. While this method detects significant allele frequency differences across an entire chromosome, we also wanted to define regions of chromosomes with significant allele frequency differences and used both a novel GAM analysis and a QTLseqr approach. In a recent study, Lima and Willet (2018), used the KS-test to map chromosomes involved in hybrid inviability between populations of copepods (*Tigriopus californicus*). They also compared PoolSeq with individual genotyping and estimated that they could detect a 0.05 allele frequency difference cutoff to Mendelian expectations (allele frequency 0.5 for diagnostic loci in F_2 hybrids). We therefore used 0.05 as an allele frequency cutoff for the GAM analysis between *Alive* and *Dead*, including a conservative approach where the 95 % confidence interval of the predicted allele frequency difference from the fitted curve should not overlap a 0.05 allele frequency difference. Here we present the false positive rate of this cutoff using a simulation approach in *R*.

Method

We assumed a chromosome length of 30 Mb containing: 100, 500 or 1,000 diagnostic loci. We had on average 412 loci per autosome and 3,057 per Z-chromosome (Table S7B). Our main goal in this study was to relate chromosomal rearrangements that differ between SWE and CAT *L. sinapis* with the evolution of hybrid inviability. Many rearrangements differentiate SWE and CAT *L. sinapis*, but the sex chromosomes are collinear (Höök et al., 2023). Thus, we only simulated an autosome.

Allele frequencies were sampled using binomial variance, representing the stochastic error due to Mendelian segregation. Since we are tracking allele frequencies of marker loci that are diagnostic between SWE and CAT, we assumed that all alleles had an expected frequency of 50 % in the F_2 offspring generation. Thus, our simulation represents chromosomes experiencing no selection. Tracking diagnostic loci, in which allelic states are shared among all parents, also means that we do not have to consider the pedigree structure. When pooling DNA, some individuals may be systematically underrepresented at all or some loci due to pipetting errors and/or other causes of differential representation. This have previously been determined to not have a major impact on estimated allele frequencies, unless sample sizes are smaller than what we used in this study (Schlötterer et al., 2014; Zhu et al., 2012). Nevertheless, we used five different constant "pool fractions". A value of 0.75 here means that a random subset (75%) of chromosomes is sampled at a certain locus. We sampled reads (with replacement) from the pool using a negative binomial distribution, following Carvalho et al., (2023). We assumed a mean read depth of 115x (conservatively at the lower end of observed means; Table S1) and a variance of read depth of 8,000 (similar to the observed variances in all pools; Table S1). Final allele frequencies per pool were then averaged by sample size (weighted average) to form the *Alive* and *Dead* groups of pools. A cubic splines GAM analysis was fit to the *Alive* vs *Dead* allele frequency difference, as explained in the Materials and methods section of the main text.

We also took the sex-averaged recombination rate into account. This is important since we expect the analytical power to generally increase with increasing recombination rate due to more across-loci variation in the binomial sampling during Mendelian segregation and recombination. Female lepidoptera are achiasmatic and thus have a recombination rate of 0 cM/Mb. While we have estimates of parental population male recombination rates (see main text), we do not know the F_1 male recombination rate. In addition, we also expect some variation in recombination rate along a chromosome. Therefore, we tested 0, 1, 2, 3 cM/Mb (sex-averaged recombination rates), but assumed a constant rate along a chromosome for simplicity. A chromosome with a varying recombination rate can in the context of this simulation can be interpreted as consisting of segments with constant recombination rates.

To make our simulations mimic our experimental setup, we measured the false positive rate per replicate of the simulation and not per locus. However, since more loci inevitably leads to a larger chance of false positives, this has to be taken into account when interpreting the results. We ran 1000 replicates for each parameter combination (n = 60). For details on the code and algorithm used in simulations, see the Github repository: https://github.com/JesperBoman/Evolution-of-hybrid-inviability-associated-with-chromosome-fusions.

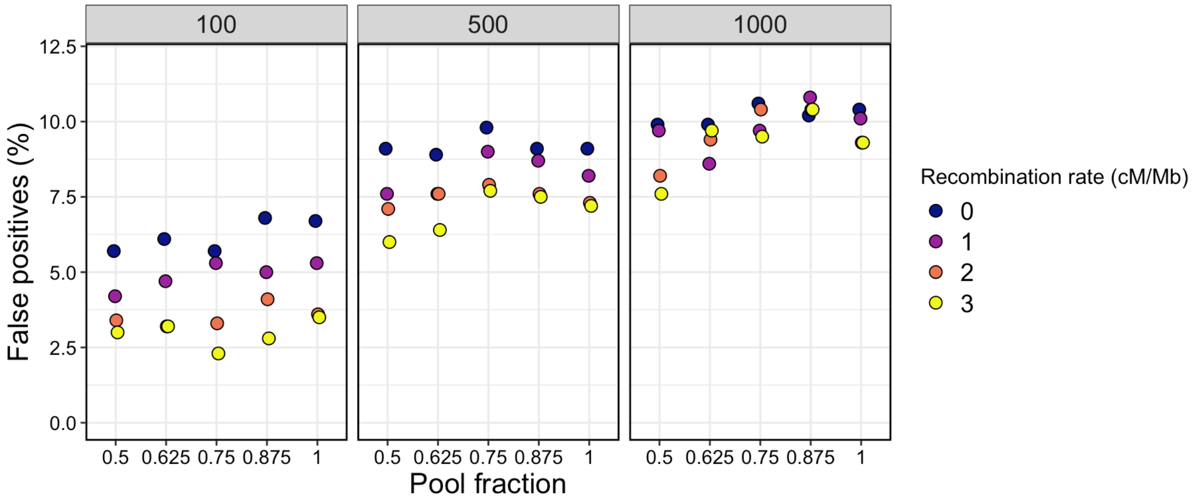

Figure ST1: False positive rate per simulated replicate when using the GAM analysis on the allele frequency difference between *Alive* and *Dead* groups of pools. The three panels represent the number of diagnostic marker loci used in each simulation replicate (n=100, 500 and 1000).

Results and discussion

The false positive rate per replicated experiment ranged from 2.3-10.8% (Figure ST1). When using 500 loci, which reflects the situation for most chromosomes in our experiment, we observed a false positive rate ranging from 6.0-9.8%. As expected, increasing the number of marker loci raises the false positive rate per simulated experiment. However, it is easy to see that it is not a simple linear relationship and thus increasing the number of marker loci decreases the false positive rate per locus (Figure ST1). The false positive rate varied relatively little with

pool fraction, indicating that this parameter has little effect for the sample sizes used in our *Alive* vs *Dead* comparison (Figure ST1).

When using 100 and 500 loci, we obtained lower false positive rates with increasing recombination rates. The reason for this that a low recombination rate creates a stronger among-locus correlation in the binomial segregation variance, which is broken by recombination events. A stronger among-locus correlation leads to larger chromosomal blocks with more consistent allele frequency differences, which increases the false positive rate. However, the difference in false positive rate between 0 and 3 cM/Mb is not large enough to account for the lower parental male recombination rate we observed in candidate regions (see Figure S7). A conservative assumption is therefore that 4 of the 37 observed hybrid inviability candidate regions are false positives (10.8%). Based only on the false positive rate for different categories of recombination in our simulations, there is a 33 % higher chance that each of those 4 candidate regions has recombination rate 0 cM/Mb instead of 3 cM/Mb. However, as can be seen in Figure S7 and in Näsvall et al., (2023), a much larger fraction of the genome has a sex-average recombination rate of 3 cM/Mb or more (6< cM/Mb when using male-specific rates). Thus, based on genomic composition of recombination rates alone, it is not likely that lower recombination rates at candidate regions are driven by false positives. Moreover, while recombination rates in F_1 hybrid males are certainly shifted due to the many rearrangements, they are not massively reduced. This can be deduced from the fact that most rearranged chromosomes have dynamic shifts in allele frequency difference between *Alive* and *Dead* along chromosomes (see Figure 2 of. the main manuscript). Such shifts are not expected if e.g. a rearranged chromosome has a recombination rate of 0 cM/Mb, since the entire chromosome should then have a similar allele frequency difference. In conclusion, the simulations performed suggest that statistical artifacts do not explain the association between recombination rates, chromosomal rearrangements and hybrid inviability candidate regions.

While investigating the true positive and false negative rate of the GAM analysis using simulations is beyond the scope of the current study, we can make certain predictions based on the GAM analysis we employed here. Using an allele frequency cutoff of 0.05 in the *Alive* and *Dead* comparison we can detect two-locus double-recessive lethal BDMIs (Figure ST2) that are expected to have an allele frequency difference of 0.0625 between *Alive* and *Dead* at each locus. To understand this, if we assume free recombination, the haplotype *aabb* is formed in 1/16 of all F_2 hybrid offspring. Those 1/16 succumb to the incompatibility and thus form part of the *Dead* group of pools. By definition, they are also then excluded from the *Alive* group of pools. This leads to an opposite but equal allele frequency shift from the Mendelian expectation in both groups of pools. If we would have compared to Mendelian expectations (allele frequency 0.5), then two-locus double-recessive BDMIs would not be detectable. If we assume a more complex dominance relationship where F_1 hybrids (haplotype *AaBb*) are viable but all haplotypes unique to the F_2 generation are lethal, then the expected allele frequency difference at both loci are 0.27 (Figure ST2C).

A.

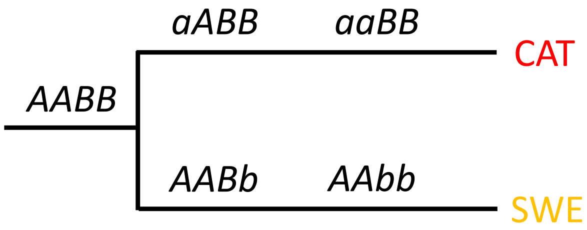

B. Two-locus double-recessive Bateson-Dobzhansky-Muller incompatibility

Locus 1 (L1)	Locus 2 (L2)
A – SWE, ancestral	B – CAT, ancestral
a – CAT, derived	b – SWE, derived

→

	BB	Bb	bb
AA	AABB 1/16	AABb 2/16	AAbb 1/16
Aa	AaBB 2/16	AaBb 4/16	Aabb 2/16
aa	aaBB 1/16	aaBb 2/16	aabb 1/16

→

	Alive		*Dead*	
	L1	*L2*	*L1*	*L2*
SWE	0.53	0.47	0.47	0.53
CAT	0.47	0.53	0.53	0.47

C. Two-locus complex recessive Bateson-Dobzhansky-Muller incompatibility

Locus 1 (L1)	Locus 2 (L2)
A – SWE, ancestral	B – CAT, ancestral
a – CAT, derived	b – SWE, derived

→

	BB	Bb	bb
AA	AABB 1/16	AABb 2/16	AAbb 1/16
Aa	AaBB 2/16	AaBb 4/16	Aabb 2/16
aa	aaBB 1/16	aaBb 2/16	aabb 1/16

→

	Alive		*Dead*	
	L1	*L2*	*L1*	*L2*
SWE	0.64	0.36	0.36	0.64
CAT	0.36	0.64	0.64	0.36

Figure ST2: Examples of Bateson-Dobzhansky-Muller incompatibilities specific for F_2+ hybrid generations. (A) A cladogram showing the mutations and substitutions at two loci: locus 1 and locus 2. (B) and (C) shows fitness diagrams and haplotype frequencies in the F_2 cohort. In (B) and (C), green boxes represent viable haplotypes and orange boxes represent lethal (completely inviable) haplotypes. Rightmost tables in (B) and (C) show the allele frequencies after selection.

Text S2. Relationship between other types of chromosomal rearrangements and hybrid inviability candidate regions

In the main text, we considered simple chromosomal rearrangements, i.e. fissions in the CAT population or fusions in the SWE population, resulting in a 2:1 homologous chromosome number ratio for the CAT:SWE population pair. Previous analyses have shown that multiple complex chromosome chain rearrangements are segregating within and between the SWE and CAT *L. sinapis* populations (Höök et al., 2023). In addition, *Brenthis* butterflies show reduced gene flow at complex rearrangements (Mackintosh et al., 2023). We therefore assessed if chromosomes involved in complex rearrangements (chromosomes 6, 13, 21, 22, 26, 30) were enriched in candidate regions. None of the chromosomes involved in complex rearrangements contained any candidate regions, which was significantly fewer than expected by chance (odds ratio = 0, $p < 0.009$).

Chromosomal inversions are prime examples of rearrangements that can reduce the crossover rate of heterokaryotypes. We characterized inversions between the two populations with whole-genome alignments of chromosome-level assemblies of CAT and SWE males. The analysis revealed 20 inverted regions between the SWE and the CAT reference. The length of the inversions ranged from 12.6 to 616.4 kb and five of the inversions intersected with hybrid inviability candidate regions. This was 1.41-fold higher than the random expectation, but not statistically significant ($p = 0.526$).

Supplementary references

Carvalho, J., Faria, R., Butlin, R. K., & Sousa, V. C. (2023). poolHelper: An R package to help in designing Pool-Seq studies. *Methods in Ecology and Evolution*, *14*(9), 2300–2307. https://doi.org/10.1111/2041-210X.14185

Höök, L., Näsvall, · K, Vila, · R, Wiklund, · C, Backström, · N, Backström, N., Näsvall, K., & Vila, R. (2023). High-density linkage maps and chromosome level genome assemblies unveil direction and frequency of extensive structural rearrangements in wood white butterflies (Leptidea spp.). *Chromosome Research*, *31*(1), 1–23. https://doi.org/10.1007/S10577-023-09713-Z

Lima, T. G., & Willett, C. S. (2018). Using Pool-seq to Search for Genomic Regions Affected by Hybrid Inviability in the copepod T. californicus. *Journal of Heredity*, *109*(4), 469–476. https://doi.org/10.1093/jhered/esx115

Mackintosh, A., Vila, R., Laetsch, D. R., Hayward, A., Martin, S. H., & Lohse, K. (2023). Chromosome Fissions and Fusions Act as Barriers to Gene Flow between Brenthis Fritillary Butterflies. *Molecular Biology and Evolution*, *40*(3), 2022.10.30.514431. https://doi.org/10.1093/molbev/msad043

Näsvall, K., Boman, J., Höök, L., Vila, R., Wiklund, C., & Backström, N. (2023). Nascent evolution of recombination rate differences as a consequence of chromosomal rearrangements. *PLOS Genetics*, *19*(8), e1010717. https://doi.org/10.1371/JOURNAL.PGEN.1010717

Schlötterer, C., Tobler, R., Kofler, R., & Nolte, V. (2014). Sequencing pools of individuals — mining genome-wide polymorphism data without big funding. *Nature Reviews Genetics*, *15*(11), 749–763. https://doi.org/10.1038/nrg3803

Zhu, Y., Bergland, A. O., González, J., & Petrov, D. A. (2012). Empirical Validation of Pooled Whole Genome Population Re-Sequencing in Drosophila melanogaster. *PLOS ONE*, *7*(7), e41901. https://doi.org/10.1371/JOURNAL.PONE.0041901
